# Epstein–Barr virus infection exacerbates ulcerative colitis by driving macrophage pyroptosis via the upregulation of glycolysis

**DOI:** 10.1093/pcmedi/pbaf002

**Published:** 2025-01-21

**Authors:** Chunxiang Ma, Kexin Chen, Lili Li, Mingshan Jiang, Zhen Zeng, Fang Yin, Jing Yuan, Yongbin Jia, Hu Zhang

**Affiliations:** Department of Gastroenterology, West China Hospital, Sichuan University, Chengdu 610041, China; Centre for Inflammatory Bowel Disease, West China Hospital, Sichuan University, Chengdu 610041, China; Lab of Inflammatory Bowel Disease, Frontiers Science Center for Disease-Related Molecular Network, West China Hospital, Sichuan University, Chengdu 610041, China; Department of Gastroenterology, West China Hospital, Sichuan University, Chengdu 610041, China; Centre for Inflammatory Bowel Disease, West China Hospital, Sichuan University, Chengdu 610041, China; Lab of Inflammatory Bowel Disease, Frontiers Science Center for Disease-Related Molecular Network, West China Hospital, Sichuan University, Chengdu 610041, China; Department of Gastroenterology, West China Hospital, Sichuan University, Chengdu 610041, China; Centre for Inflammatory Bowel Disease, West China Hospital, Sichuan University, Chengdu 610041, China; Lab of Inflammatory Bowel Disease, Frontiers Science Center for Disease-Related Molecular Network, West China Hospital, Sichuan University, Chengdu 610041, China; Department of Gastroenterology, West China Hospital, Sichuan University, Chengdu 610041, China; Centre for Inflammatory Bowel Disease, West China Hospital, Sichuan University, Chengdu 610041, China; Lab of Inflammatory Bowel Disease, Frontiers Science Center for Disease-Related Molecular Network, West China Hospital, Sichuan University, Chengdu 610041, China; Department of Gastroenterology, West China Hospital, Sichuan University, Chengdu 610041, China; Centre for Inflammatory Bowel Disease, West China Hospital, Sichuan University, Chengdu 610041, China; Lab of Inflammatory Bowel Disease, Frontiers Science Center for Disease-Related Molecular Network, West China Hospital, Sichuan University, Chengdu 610041, China; Department of Gastroenterology, West China Hospital, Sichuan University, Chengdu 610041, China; Centre for Inflammatory Bowel Disease, West China Hospital, Sichuan University, Chengdu 610041, China; Lab of Inflammatory Bowel Disease, Frontiers Science Center for Disease-Related Molecular Network, West China Hospital, Sichuan University, Chengdu 610041, China; Department of Gastroenterology, West China Tianfu Hospital, Sichuan University, Chengdu 610213, China; Lab of Inflammatory Bowel Disease, Frontiers Science Center for Disease-Related Molecular Network, West China Hospital, Sichuan University, Chengdu 610041, China; Department of Gastroenterology, West China Hospital, Sichuan University, Chengdu 610041, China; Centre for Inflammatory Bowel Disease, West China Hospital, Sichuan University, Chengdu 610041, China; Lab of Inflammatory Bowel Disease, Frontiers Science Center for Disease-Related Molecular Network, West China Hospital, Sichuan University, Chengdu 610041, China; Department of Gastroenterology, West China Tianfu Hospital, Sichuan University, Chengdu 610213, China

**Keywords:** Epstein-Barr virus, ulcerative colitis, macrophages, pyroptosis; glycolysis

## Abstract

**Background:**

Epstein–Barr virus (EBV) infection is associated with clinical symptoms, treatment response, need for surgical intervention, and an enhanced likelihood of lymphoma among patients with ulcerative colitis (UC). However, existing studies have primarily concentrated on the epidemiological and clinical associations between EBV and UC, leaving the mechanisms by which EBV exacerbates colitis poorly understood.

**Methods:**

Clinical specimens of UC patients with EBV infection and a mouse model of dextran sulfate sodium-induced colitis with concurrent murine γ-herpesvirus 68 (MHV-68) infection were utilized to investigate the relationship between EBV infection and macrophage pyroptosis. *In vivo*, adoptive transfer of MHV-68-induced macrophages and macrophage depletion were performed to elucidate the underlying mechanisms. *In vitro*, myeloid leukemia mononuclear cells of human (THP-1) and macrophages derived from mouse bone marrow (BMDMs) were stimulated with EBV and MHV-68, respectively, to assess macrophage pyroptosis and glycolysis.

**Results:**

EBV-induced activation of macrophage pyroptosis was positively correlated with clinical disease activity in UC patients. Furthermore, MHV-68 infection activated pyroptosis by upregulating gasdermin D, NLRP3, interleukin-1β, and interleukin-18 in colonic tissues and peritoneal macrophages of mice with colitis. *In vitro*, EBV and MHV-68 also mediated activation of pyroptosis in human THP-1 cells and mouse BMDMs, respectively. Additionally, the adoptive transfer of MHV-68-induced BMDMs aggravated murine colitis, whereas macrophage depletion attenuated MHV-68-induced intestinal injury. Mechanistically, MHV-68 promoted macrophage pyroptosis by upregulating glycolysis, while the glycolysis inhibitor, 2-deoxy-D-glucose, blocked this process *in vitro*.

**Conclusion:**

EBV infection exacerbates UC by driving macrophage pyroptosis through upregulation of glycolysis, indicating a potential therapeutic approach to mitigate EBV-induced intestinal inflammation.

## Graphical Abstract

**Graphical Abstract:**
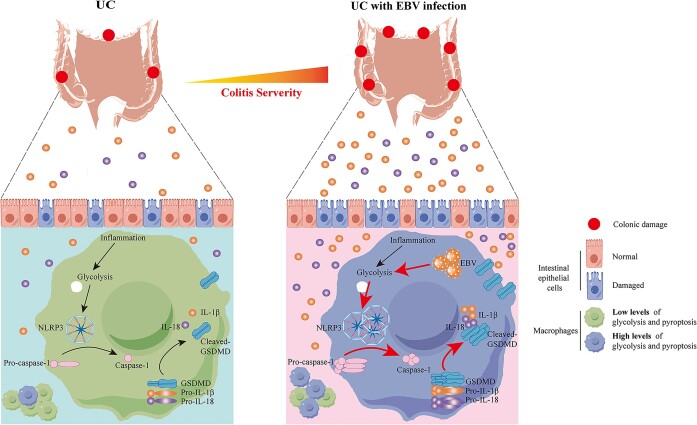
Epstein-Barr virus infection induces the upregulation of glycolysis in intestinal macrophages, which subsequently activates Gasdermin D, NLRP3, interleukin-1β, and interleukin-18 in macrophages within colonic tissues. The release of these pro-inflammatory cytokines results in intestinal barrier dysfunction and exacerbates ulcerative colitis.

## Introduction

Ulcerative colitis (UC) is an ongoing form of inflammation of the gastrointestinal tract that is characterized by recurring inflammation and ulcer formation [1, 2]. Current medical therapies for UC predominantly focus on alleviating the inflammatory process by employing a range of immunosuppressive medications, encompassing corticosteroids, immunomodulators, and biological therapies [3–5]. Although these drugs effectively control UC symptoms, their immunosuppressive properties significantly intensify the vulnerability to opportunities for infection, including Epstein–Barr virus (EBV) [6, 7]. Researchers have observed elevated EBV DNA and RNA levels in the mucosa of the colon of individuals with UC. These levels are closely linked to disease severity and inflammatory markers in patients [8–10]. Some studies suggest that active EBV infection in patients with UC is intricately linked to clinical manifestations, therapeutic responses, requirement for surgical intervention, and increased lymphoma risk [11–13]. However, these studies predominantly focused on the epidemiological and clinical correlations between EBV and UC. The mechanisms by which EBV infection contributes to UC pathogenesis, particularly its role in initiating or sustaining mucosal inflammation, remain to be fully elucidated.

Inflammation-induced pyroptosis is a carefully orchestrated phenomenon of intentional cell suicide regulated by inflammasomes, notably theNOD-like receptor family pyrin domain containing 3 (NLRP3) inflammasome [14]. When triggered by microbial-associated molecular patterns, alarmins, or various stress-inducing factors, the NLRP3 inflammasome autoactivates precursor caspase-1 to generate mature caspase-1 [15]. Subsequently, this mature enzyme cleaves and activates precursor interleukin-1β (IL-1β), precursor interleukin-18 (IL-18), and gasdermin D (GSDMD) [15]. Cleaved-GSDMD subsequently inserts cell membrane holes for mature IL-1β and IL-18 spilling over from the cytoplasm, thereby amplifying the inflammatory response of inflammation [15]. In recent research, pyroptosis has gained attention for its role in exacerbating mucosal inflammation in UC [16, 17]. Studies analyzing genetic polymorphisms in peripheral blood DNA from UC patients have identified associations between polymorphisms in NLRP3 and IL-18 and susceptibility to UC [18, 19]. Assessments of protein and RNA transcript levels in the colonic areas of individuals with UC also revealed substantially higher expression of pro-IL-18 and fully mature IL-18, particularly in inflamed colonic tissues, compared to healthy controls [20]. Enzyme-linked immunosorbent assay (ELISA) of serum from UC patients have equally revealed positive correlations between NLRP3 levels and disease severity [21]. Furthermore, mouse models have underscored the pivotal role of activated pyroptosis in colitis. When treated with dextran sulfate sodium (DSS), mice with knockout of caspase-1 and NLRP3 showed considerable improvement in colitis. This improvement was demonstrated by a reduction in weight loss, preservation of colon length, mitigation of intestinal pathology, and a decrease in the levels of colitis-associated inflammatory markers [22]. Meanwhile, administration of pyroptosis inhibitors can effectively mitigate the effects of colitis caused by DSS [21]. Collectively, excessive pyroptosis can perpetuate immoderate and sustained inflammatory responses and thus play an essential role in the progression of UC.

Glycolysis, a central metabolic pathway that converts glucose into pyruvate, plays a pivotal role not only in energy production but also in regulating immune cell functions under both physiological and pathological conditions. Emerging evidence suggests that glycolysis is significantly upregulated in inflammatory conditions, contributing to the pathogenesis of UC. In UC, heightened glycolytic activity has been implicated in driving pro-inflammatory responses by enhancing the activation and effector functions of immune cells, such as macrophages and T cells, within the intestinal microenvironment [23–25]. Furthermore, EBV infection has been shown to induce glycolysis in infected cells, which promotes the production of proinflammatory cytokines and exacerbates tissue inflammation [26–28]. However, the mechanistic link between glycolysis and EBV-induced intestinal inflammation remains unclear.

Macrophages, as key components of intestinal microenvironment homeostasis, are considered major targets of intestinal pyroptosis and significantly contribute to UC pathogenesis. Studies conducted *in vitro* have demonstrated that treatment of macrophages with DSS stimulates the synthesis of NLRP3, together with the generation of IL-1β in a caspase-1-driven process [22]. These findings align with observations in humans, where CD14^+^ blood monocytes/macrophages from peripheral blood of patients with UC secreted elevated levels of IL-1β after lipopolysaccharide stimulation [29]. Additionally, data from single-cell analyses further reinforced the conclusion that macrophages are the primary sites of activation for intestinal pyroptosis in UC patients [29, 30]. Notably, recent studies have established a connection between pyroptosis and EBV-associated diseases. In patients with acute EBV exposure, there has been a notable observation of elevated serum levels of IL-18, showing an upward link to disease severity [31]. Furthermore, upon stimulation with EBV peptide antigens, whole blood cells from EBV-positive patients with multiple sclerosis show increased secretion of IL-1β compared to those from healthy individuals [32]. EBV infection even directly activates the pyroptosis signaling pathway in human monocytes [33]. However, it remains unclear whether EBV infection exacerbates intestinal inflammation in UC by activating the pyroptosis signaling pathway in intestinal macrophages.

Based on existing evidence linking EBV infection to macrophages, pyroptosis, glycolysis, and inflammatory responses, we hypothesize that EBV exacerbates UC inflammation by inducing macrophage pyroptosis through glycolysis activation. This study aims to elucidate whether EBV infection exacerbates intestinal inflammation in UC through the activation of macrophage pyroptosis, and to uncover the mechanisms involved. Our results proved a strong association between the activation of macrophage pyroptosis caused by EBV and the clinical manifestation of disease activity of UC. Furthermore, the pro-pyroptotic effect of EBV in macrophages was shown to be dependent on glycolysis activation in our findings. This study expands the understanding of the detrimental effects of EBV on the progression of UC and suggests potential therapeutic and preventive strategies for EBV-associated inflammation.

## Materials and methods

### Patients and sample collection

EBV-encoded RNA (EBER) throughout the colon has been studied to determine EBV infection, as identified through EBER-*in situ* hybridization (EBER-ISH). Specifically, tissues from colonic biopsies of patients with UC were de-paraffinised, rehydrated, and permeabilised with proteinase K, followed by overnight hybridization at 37 °C with digoxigenin-labelled EBER probes. After incubation with horseradish peroxidase-conjugated anti-digoxigenin antibody, EBER-positive cells were identified by diaminobenzidine chromogenic staining, exhibiting brown-stained nuclei. Patients with positive EBER-ISH results were categorized as having intestinal EBV coinfection, whereas those with negative results were classified as non-infected. Subsequently, colonic tissues from UC patients with and without EBV infection were exposed to primary antibodies targeting anti-CD68, anti-IL-18, anti-GSDMD, or anti-IL-1β on the first day (for detailed data see supplementary Table 1 in the online supplementary material), and secondary antibodies were added on the second day. Images were acquired using the VS200 whole-slide illumination device (Olympus). We evaluated the relationship between the abundance of markers involved in macrophage pyroptosis and disease activity in patients with UC. This was performed using Pearson correlation analysis. The ethical committee of West China Hospital granted authorization for the collection of colonic samples and analysis of clinical data (Approval Number: 2023–22).

### Animal experiments

The mice (C57BL/6, male), obtained from GemPharmatech Co., Ltd. (Chengdu, China) at 6–8 weeks of age, were housed in the BSL-2 animal center at West China Hospital. All mice were randomized into experimental groups and acclimatized for 7 days before beginning the experimental protocol. Mice were provided with an oral 2% (w/v) solution containing DSS, with a molecular weight ranging between 36 000 and 50 000 Da, mixed in their drinking water for an interval of 5 days. Subsequently, they were returned to regular water for an additional period of 3 days to trigger colitis. To simulate colitis concurrent with MHV-68 infection, mice were intraperitoneally injected with 200 µl of murine γ-herpesvirus 68 (MHV-68) at an injection dose of 1 × 10^6^ plaque-forming units (PFU)/ml before DSS administration. To ensure the systemic dissemination of the macrophages via the bloodstream, adoptive transfer procedures involved stimulating bone marrow-derived macrophages (BMDMs) with MHV-68 or phosphate-buffered saline *in vitro* for 12 h prior to tail vein injection. On the third day of DSS treatment, 1 × 10^6^ BMDMs were delivered to DSS-treated mice via tail vein injection in 200 µl of PBS. For macrophage depletion, 200 µl of clodronate liposome (LIPO; Yeasen, China) was intravenously injected into MHV-68-infected mice 1 day before and 3 days after DSS administration. Daily weight measurements of the mice were taken, with continuous assessment of the disease activity index (DAI) to track disease progression throughout the experimental period, as previously documented [34]. The animals were euthanized on the ninth day, and samples of colonic tissues, lamina propria mononuclear cells (LPMCs), and peritoneal macrophages were procured. Histological scoring was performed according to depth of the ulcer (0–4), extent of the ulcer (0–4), presence of inflammation (0–4), extent of inflammation (0–4), and location of fibrosis (0–4) [35]. Endoscopic scoring was performed as previously described [36]. The Animal Ethics Committee of West China Hospital granted authorization for all animal experiments in this research (Approval Number: 2023–22).

### Cell culture

Myeloid leukemia mononuclear cells of human (THP-1) were transformed into adherent macrophages by treatment with phorbol 12-myristate 13-acetate (PMA; 100 nM) for 24 h. BMDMs were treated with mouse macrophage colony-stimulating factor (M-CSF) to drive differentiation, whereas 2-deoxy-D-glucose (2-DG) was used to inhibit glycolysis. To obtain peritoneal macrophages, 10 ml of cold PBS was injected into the abdominal cavity of mice, coupled with a 3-min abdominal massage to obtain peritoneal lavage fluid. Cells were pelleted from the lavage fluid after centrifugation for 5 min at 1500 rpm and subsequently used for further experiments.

### Isolation of LPMCs

Colonic tissues were minced and digested using a solution prepared for predigestion with ethylenediaminetetraacetic acid, dithiothreitol, fetal bovine serum, and 4-(2-hydroxyethyl)-1-piperazineethanesulfonic acid. After the first filtration, tissue fragments underwent a second digestion with a solution containing DNase, collagenase VIII, and FBS. LPMCs were gathered from the obviously stratified area between 40% and 80% Percoll after a second filtration and centrifugation at 2200 revolutions per minute for 20 min. All reagents used in this experiment were purchased from Solarbio (Beijing, China). For flow cytometry analysis, FITC-CD11b (Biolegend, USA), Alexa Fluor 700-CD45 (Biolegend, USA), and Brilliant Violet 605-F4/80 (Biolegend, USA) were used to stain LPMCs.

### Virus preparation

The culture supernatant from the B95-8 cells was ultracentrifuged at 100 000 × g to isolate EBV. EBV was suspended in PBS and stored at −80 °C. BHK-21 cells were exposed to 30 µl of virus suspension containing 1 × 10^5^ PFU of MHV-68 and cultured for 2 days. Afterwards, the BHK-21 cells and culture supernatant were collected and subjected to two freeze–thaw cycles. The mixture was then centrifuged and filtered to eliminate cell debris. The clarified supernatant was divided into smaller portions and stored at −80 °C. All experiments involving EBV and MHV-68 were conducted in the BSL-2 laboratory.

### Western blot

A specialized buffer from Solarbio, China, was used to thoroughly lyse colon tissues or cell lines. This was followed by loading 25 µg of total protein per well onto an sodium dodecyl sulfate-polyacrylamide gel electrophoresis apparatus and depositing it onto polyvinylidene fluoride membranes. After blocking with a solution containing non-fat powdered milk in tris-buffered saline with Tween 20, the membranes were incubated at low temperature for an extended period with primary antibodies (detailed data is given in Supplementary Table 1). On the second day, the membranes were coated with secondary antibodies bound with HRP (1:10 000, BioXcell, China) for 1 h at room temperature. We used a chemiluminescence imaging system from Tanon, China, to identify the immunoreactive bands and then analyzed them using ImageJ (version 1.53).

### Quantitative real-time PCR

RNA purification from colonic tissues or cell lines was performed using TRIzol Reagent. Reverse transcription-based cDNA formation was executed with UnionScript First-strand cDNA Synthesis Mix, which is manufactured by Beijing Genesand Biotech in China. Quantitative reverse transcription polymerase chain reaction (RT-qPCR) Kit (Abclone, China) with SYBR Green was used to conduct real-time qPCR. The complete primer list is provided in supplementary Table 2 (see online supplementary material). The mRNA levels of target genes were calculated in relation to glyceraldehyde 3-phosphate dehydrogenase (GAPDH) expression. Quantification was performed using the 2^−ΔΔCt^ methodology.

### Statistical analysis

The data are shown as mean ± standard error (SEM), comparisons between two groups were made using Student's t-test, and comparisons among three or more groups were made using an ANOVA test. Relevance evaluations were performed using Pearson correlation analysis. The significance level was set at *P* < 0.05.

## Results

### EBV-induced intestinal macrophage pyroptosis is correlated with disease activity of UC among patients

**Figure 1.**
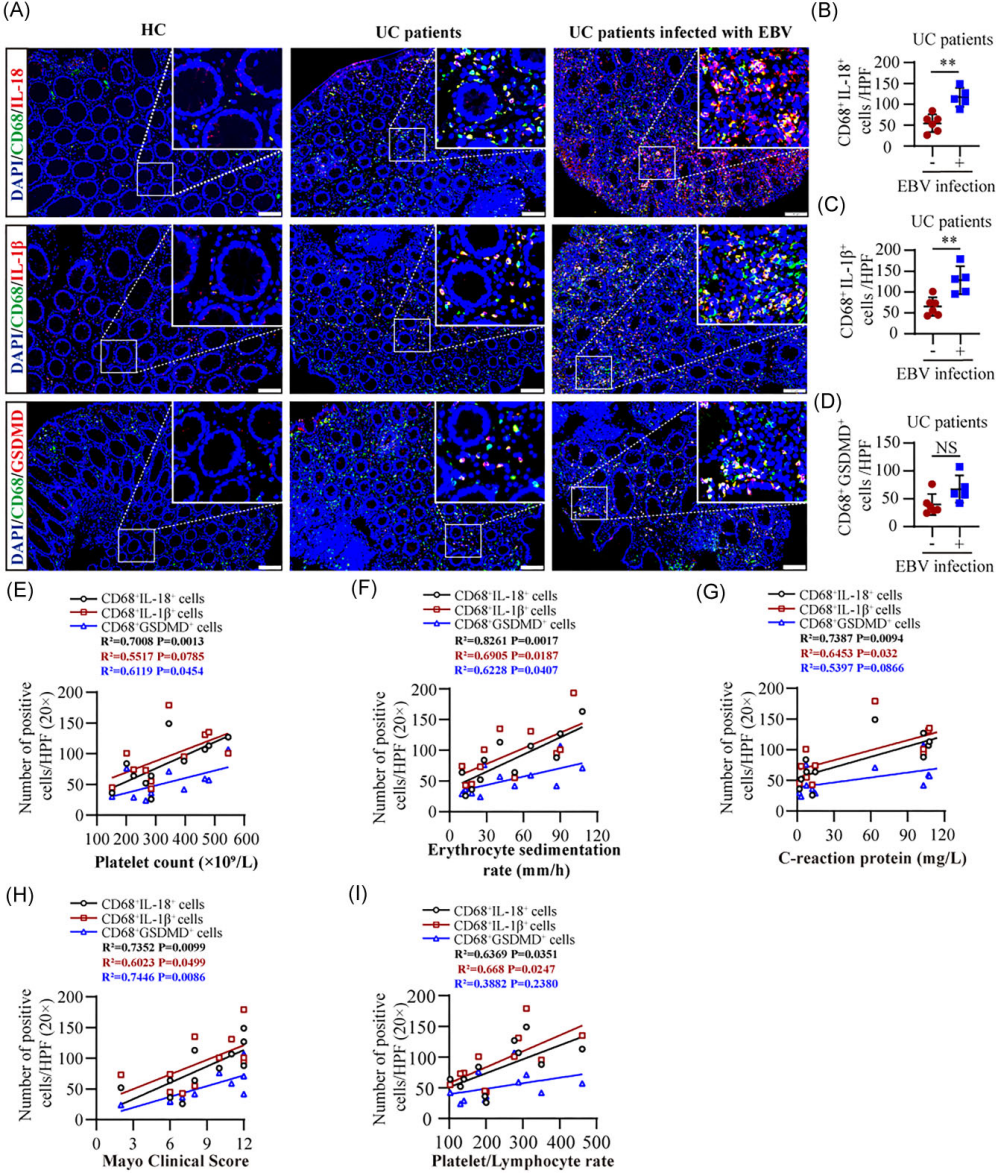
EBV-induced intestinal macrophage pyroptosis is correlated with disease activity of UC patients. (**A**) Representative immunofluorescence staining of colonic tissues showing cell nucleus (blue), CD68 (green), IL-18 (red), IL-1β (red) or GSDMD (red). (**B**–**D**) Quantification of double-positive cells per high-power field (HPF, 20×) in colonic tissues from UC patients with (*n* = 5) and without (*n* = 6) EBV infection. Panels show the number of CD68 and IL-18 (B), CD68 and IL-1β (C), and CD68 and GSDMD (D) double-positive cells. Correlation analysis of CD68 and IL-18, CD68 and IL-1β, and CD68 and GSDMD double-positive cells per HPF with disease activity indicators: PLT (**E**), ESR (**F**), CRP (**G**), Mayo Clinic Activity Score (**H**), and PLR (**I**) in UC patients with (*n* = 5) and without (*n* = 6) EBV infection. Scale bars corresponds to 100 μm (A). **P ≤ 0.01. NS, Not significant.

To clarify the connection between pyroptosis of intestinal macrophages and EBV infection in UC, colonic mucosa samples were collected from patients with EBER-negative and EBER-positive UC. Supplementary Fig. 1 (see online supplementary material) presents histological evidence of intestinal EBV infection through EBER-ISH, while supplementary Table 3 (see online supplementary material) provides an overview of the clinical characteristics of these patients. Dual immunofluorescence staining was utilized to co-localize the macrophage marker CD68 with pyroptosis markers IL-18, IL-1β, and GSDMD in their intestinal tissues. Our findings revealed that UC patients positive for intestinal EBER exhibited significantly higher levels of intestinal macrophage pyroptosis than those negative for intestinal EBER (Fig. 1A–D). Additionally, platelet count (PLT), erythrocyte sedimentation rate (ESR), C-reactive protein (CRP), Mayo Clinic Activity Score, and platelet/lymphocyte rate (PLR) served as reliable indicators of clinical disease activity in UC [37, 38]. To gain insight into the connection between clinical disease activity and intestinal macrophage pyroptosis among EBV-infected UC patients, clinical correlation analyses were conducted. Linear regression analysis demonstrated that the number of CD68 and IL-18 co-localized cells was significantly correlated with PLT (*R*^2^ = 0.7008, *P* = 0.0013), ESR (*R*^2^ = 0.8261, *P* = 0.0017), CRP (*R*^2^ = 0.7387, *P* = 0.094), Mayo Clinic Activity Score (*R*^2^ = 0.7352, *P* = 0.0099), and PLR (*R*^2^ = 0.6369, *P* = 0.0351) in Fig. 1E–I. Similarly, the number of CD68 and IL-1β co-localized cells exhibited correlations with PLT (*R*^2^ = 0.5517, *P* = 0.0785), ESR (*R*^2^ = 0.6905, *P* = 0.0187), CRP (*R*^2^ = 0.6453, *P* = 0.032), Mayo Clinic Activity Score (*R*^2^ = 0.6023, *P* = 0.0499), and PLR (*R*^2^ = 0.668, *P* = 0.0247) in Fig. 1E–I. Meanwhile, the number of CD68 and GSDMD co-localized cells was correlated with PLT (*R*^2^ = 0.6119, *P* = 0.0454), ESR (*R*^2^ = 0.6228, *P* = 0.0407), CRP (*R*^2^ = 0.5397, *P* = 0.0866), Mayo Clinic Activity Score (*R*^2^ = 0.7446, *P* = 0.0086), and PLR (*R*^2^ = 0.3882, *P* = 0.2380) in Fig. 1E–I. Collectively, these findings confirm that EBV infection induces intestinal macrophage pyroptosis, which is positively correlated with clinical disease activity in patients with UC.

### MHV-68 infection exacerbates DSS-induced colitis by activating pyroptosis of colonic tissues and peritoneal macrophages in mice

**Figure 2.**
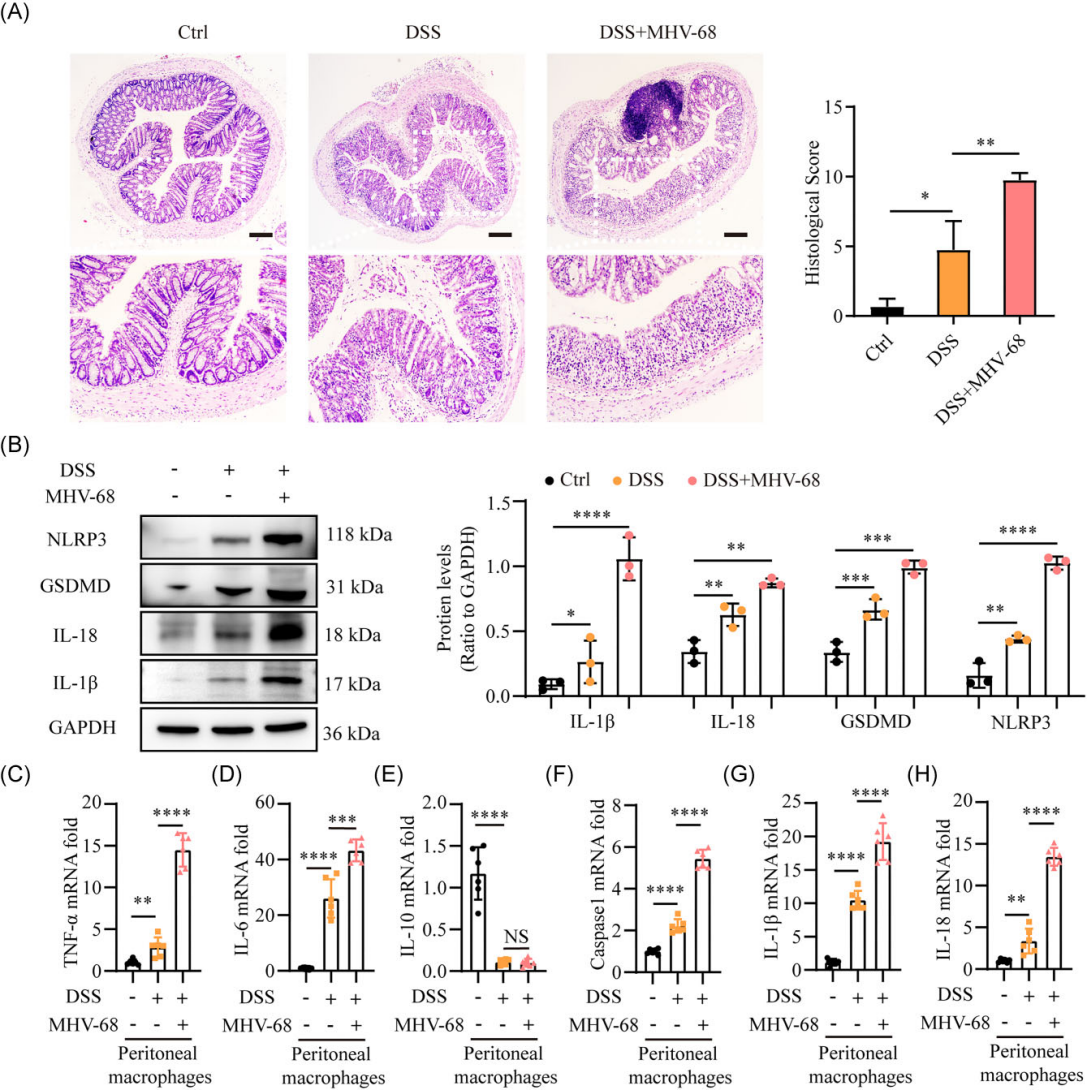
MHV-68 infection exacerbates DSS-induced colitis by activating pyroptosis of colonic tissues and peritoneal macrophages in mice. (**A**) Representative hematoxylin and eosin (H&E)-stained images of colon tissues (left) and the corresponding histopathological evaluation scores (right); *n* = 3–4. (**B**) Western blot analysis shows protein levels of NLRP3, GSDMD, IL-1β, and IL-18 of colonic tissues. Relative mRNA expression levels of (**C**) TNF-α, (**D**) IL-6, (**E**) IL-10, (**F**) caspase-1, (**G**) IL-1β, and (**H**) IL-18 in peritoneal macrophages isolated from DSS-only and DSS + MHV-68-infected groups. Scale bars in (A) represent 500 μm. *P ≤ 0.05, **P ≤ 0.01, ***P ≤ 0.001, and ^****^P ≤ 0.0001. NS, Not significant.

MHV-68, a natural γ-herpesvirus of mice, shares significant genetic and biological similarities with human EBV [39]. These similarities make MHV-68 a reliable experimental model for investigating the fundamental biology and therapeutic strategies of EBV infection. To determine whether EBV promoted colitis through the activation of intestinal macrophage pyroptosis, we intraperitoneally injected MHV-68 into mice with DSS-induced colitis to simulate EBV infection in patients with UC. Subsequently, DNA-PCR analysis confirmed the presence of the M1 gene of MHV-68 in both the spleen and colonic macrophages of these mice, verifying that MHV-68 infected the intestines of mice with DSS-induced colitis (Supplementary Fig. 2, see online supplementary material). Histological examination showed dramatic worsening of mucosal deterioration, infiltration of inflammation-related cells, and loss of crypts, indicating that intestinal inflammation in the DSS group with MHV-68 infection was far more pronounced than that observed in the DSS group (Fig. 2A). We further explored intestinal pyroptosis in MHV-68-infected mice treated with DSS to determine its role in colitis. Western blot analysis confirmed that mice infected with MHV-68 had elevated levels of NLRP3, IL-1β, GSDMD, and IL-18 based on protein expression levels in their colons (Fig. 2B). Furthermore, peritoneal macrophages were extracted from mice subjected to DSS treatment, regardless of MHV-68 infection, to determine the transcript levels of inflammatory mediators and pyroptosis indicators in mice. Infection with MHV-68 resulted in an elevated abundance of cytokines (TNF-α and IL-6) that triggered inflammation (Fig. 2C and D), an upsurge in pyroptosis-related molecules involving caspase-1, IL-1β, and IL-18 (Fig. 2F–H), along with a decrease in IL-10 concentration, an anti-inflammatory cytokine (Fig. 2E), in peritoneal macrophages. In addition, we examined the impact of MHV-68 infection on the functional polarization of intestinal macrophages in DSS-treated mice. Western blotting analysis revealed a significant upregulation of the M1 pro-inflammatory macrophage marker inducible nitric oxide synthase (iNOS) in the intestinal tissue of MHV-68-infected mice, with no detectable changes in marker CD206 associated with M2 anti-inflammatory macrophages (Supplementary Fig. 3, see online supplementary material). These findings indicate that MHV-68-induced macrophage pyroptosis and M1 polarization significantly contribute to the worsening of DSS-induced colitis.

### EBV induces pyroptosis and promotes proinflammatory cytokine production in human macrophages *in vitro*

**Figure 3.**
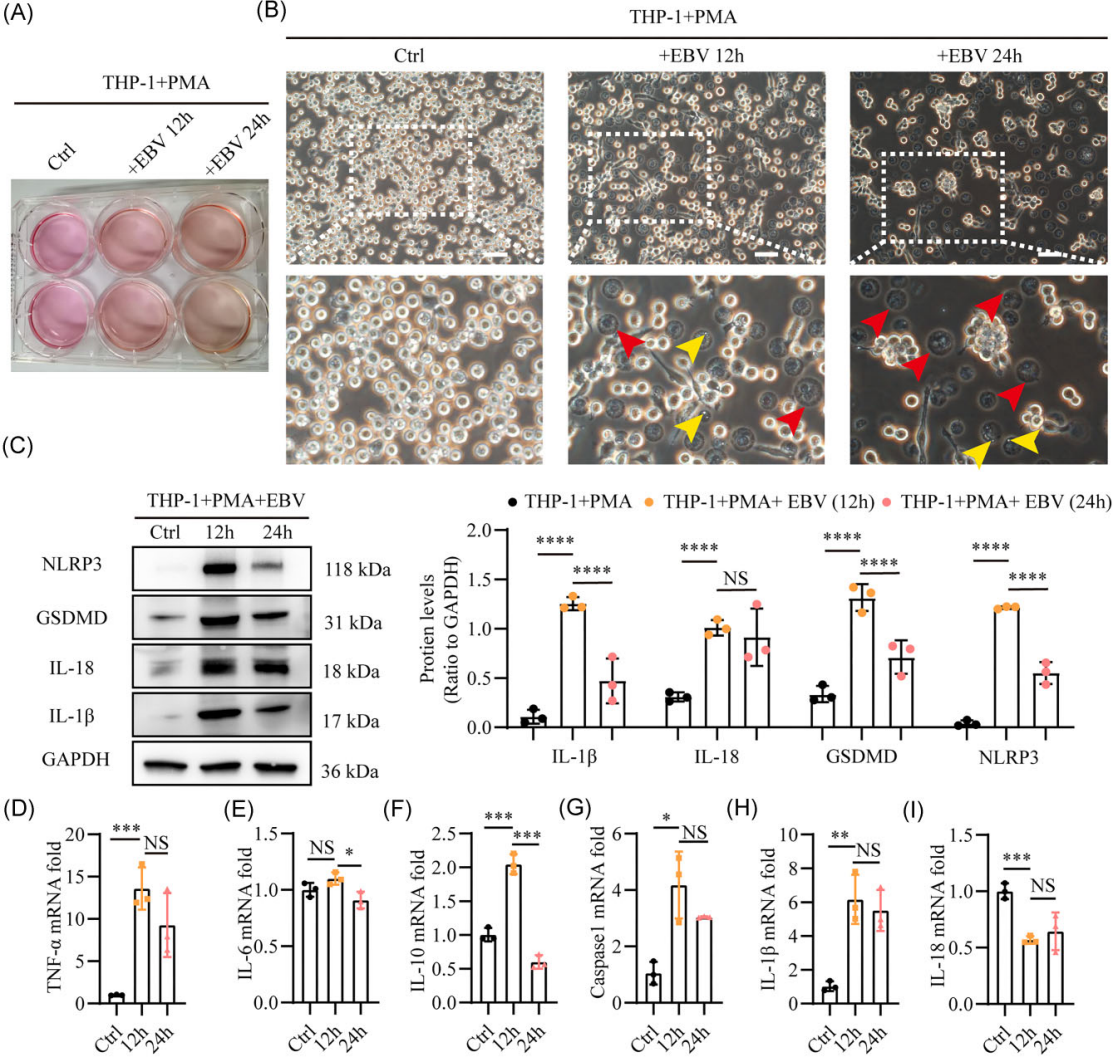
EBV induces pyroptosis and promotes proinflammatory cytokine synthesis in human macrophages *in vitro*. THP-1 cells underwent pretreatment with PMA for 24 h, followed by stimulation with EBV for 12 or 24 h. (**A**) Representative images showing color changes in the culture supernatant. (**B**) Representative light microscopy images displaying morphological changes in THP-1 cells, with red arrowheads indicating cell swelling and yellow arrowheads marking distinctive blebbing structures. (**C**) Western blot analysis of NLRP3, GSDMD, IL-18, and IL-1β protein levels associated with the pyroptosis pathway. Relative mRNA expression levels of (**D**) TNF-α, (**E**) IL-6, (**F**) IL-10, (**G**) caspase-1, (**H**) IL-1β, and (**I**) IL-18 were quantified by RT-qPCR. Scale bars in (A) represent 500 μm. *P ≤ 0.05, **P ≤ 0.01, ***P ≤ 0.001, and ^****^P ≤ 0.0001. NS, Not significant.

To verify whether EBV infection could directly activate pyroptosis in human macrophages, we differentiated THP-1 monocytes into mature macrophages through 24-h PMA treatment, followed by exposure of these cells to EBV for 12 and 24 h. Over time, the incubated supernatant of EBV-stimulated THP-1 macrophages exhibited significant yellowing (Fig. 3A). Additionally, progressive cell death was observed under light microscopy during primary EBV infection, characterized by cell aggregation, loss of transparency, and classic pyroptosis morphology, such as significant cell swelling and distinctive blebbing structures (Fig. 3B). Subsequently, we used DNA-PCR with five different primers targeting the EBER gene of EBV in THP-1 cells, confirming the presence of EBV infection (Supplementary Fig. 2B). RT-qPCR and western blot studies also verified that an elevated pyroptosis signaling pathway occurred in THP-1 cells as a result of EBV infection. This upregulation resulted in increased concentrations of GSDMD, IL-18, NLRP3, IL-1β, caspase-1, and IL-1β (Fig. 3C and G–I). Furthermore, this stimulation of pyroptosis was accompanied by elevated TNF-α and IL-6 levels, indicative of a pro-inflammatory state (Fig. 3D and E), while IL-10, an anti-inflammatory cytokine, decreased (Fig. 3F). Notably, primary EBV infection markedly modified the pyroptosis and inflammatory cytokine landscapes in human macrophages, with notable changes evident at the 12-h mark (Fig. 3C–I). Taken together, our data indicate that the exposure of human macrophages to EBV triggers pyroptosis and amplifies pro-inflammatory cytokine production.

### Adoptive transfer of MHV-68-induced BMDMs significantly exacerbates DSS-induced experimental colitis

**Figure 4.**
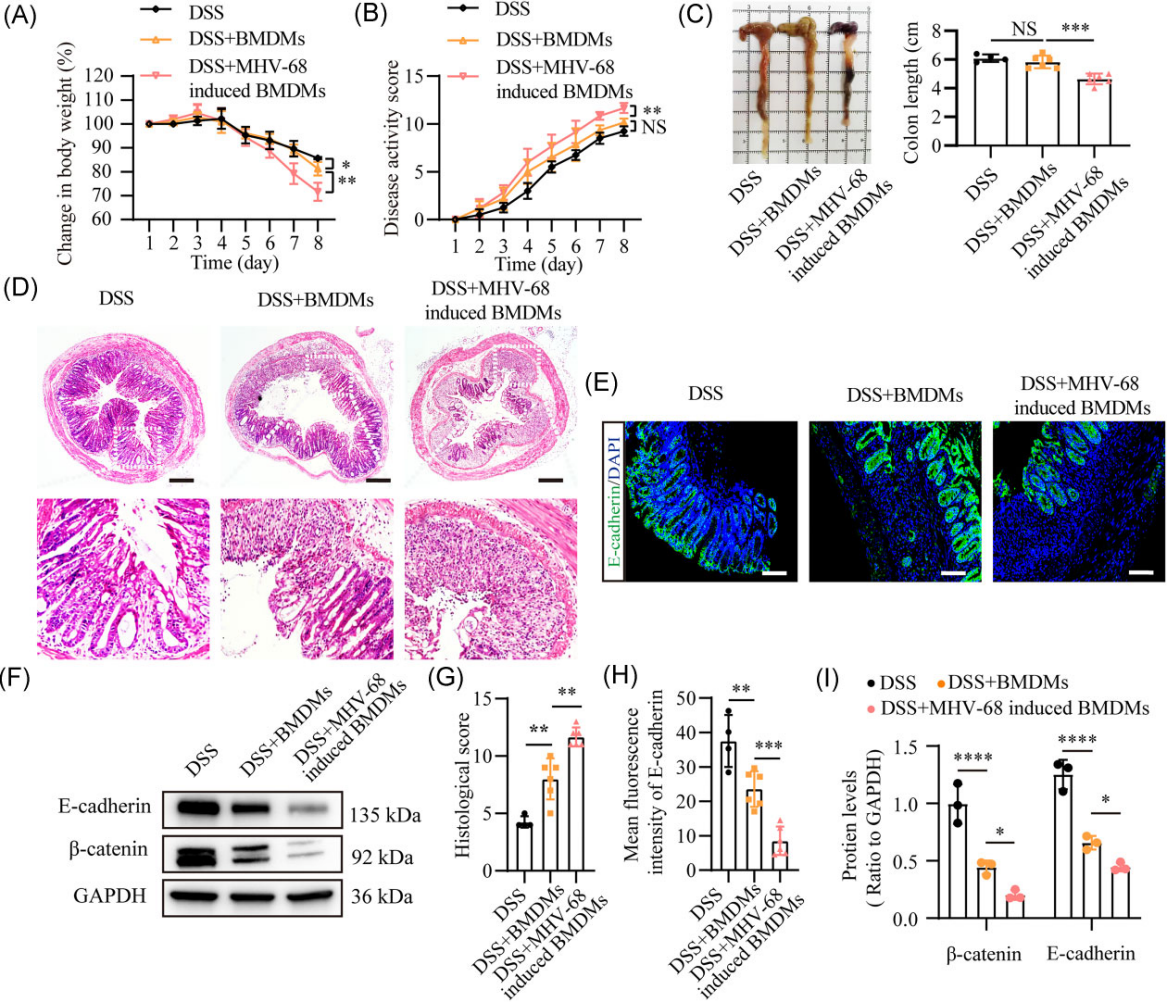
EBV Adoptive transfer of MHV-68-induced BMDMs significantly exacerbates DSS-induced experimental colitis. Colitis severity was assessed by weight loss (**A**), DAI score (**B**), colon length (**C**), representative hematoxylin and eosin (H&E)-stained colon sections (**D**), and histological scores (**G**); *n* = 4–6. Immunofluorescence analysis of E-cadherin (green) expression in colonic tissues (**E, H**) and western blot analysis of colonic E-cadherin and β-catenin levels (**F, I**) following the adoptive transfer. DAPI (blue) was used to visualize the nuclei. Scale bars represent 500 μm (D) and 100 μm (E). *P ≤ 0.05, **P ≤ 0.01, ***P ≤ 0.001, ^****^P ≤ 0.0001. NS, Not significant.

To investigate whether murine MHV-68 virus can activate pyroptosis in murine macrophages, we stimulated BMDMs with MHV-68 for 12 and 24 h, followed by RT-qPCR to confirm MHV-68-infected BMDMs and western blotting to detect pyroptosis-related proteins. The data revealed that MHV-68 indeed infected BMDMs and effectively boosted the generation of pyroptosis proteins, including GSDMD, NLRP3, IL-18, and IL-1β in BMDMs, with the most pronounced effect observed at 12 h post-stimulation (Supplementary Fig. 4, see online supplementary material). This effect was comparable to the stimulatory effect of pyroptosis induced by EBV infection in human macrophages. Furthermore, to assess the direct physiological effects of MHV-68-induced macrophages on colitis, we treated BMDMs with MHV-68 or PBS for 12 h *in vitro*, followed by adoptive transfer into mice that had been exposed to 2% DSS water for 3 days. The mice continued to receive 2% DSS water for an additional 2 days before being switched to regular drinking water for 3 days (supplementary Fig. 5, see online supplementary material). The mice were euthanized on the ninth day, and measurements were taken of their body weight, DAI scores, colon length, and histopathological alterations of the colon to assess the severity of intestinal damage. As shown by pronounced weight loss, elevated DAI scores, elevated histopathological scores, and shortened colon length, transfer of MHV-68-induced BMDMs significantly worsened experimental colitis induced by DSS (Fig. 4A–D and G). Immunofluorescence and western blot analyses indicated that, compared to mice receiving PBS-induced BMDMs, those transferred with MHV-68-induced BMDMs exhibited greater intestinal barrier disruption, as indicated by reduced levels of β-catenin and E-cadherin (Fig. 4E and F, H and I). These findings demonstrate that adoptive transfer of MHV-68-induced macrophages aggravates DSS-induced colitis.

### Macrophage depletion alleviates intestinal inflammation exacerbated by MHV-68 infection in DSS-induced colitis

**Figure 5.**
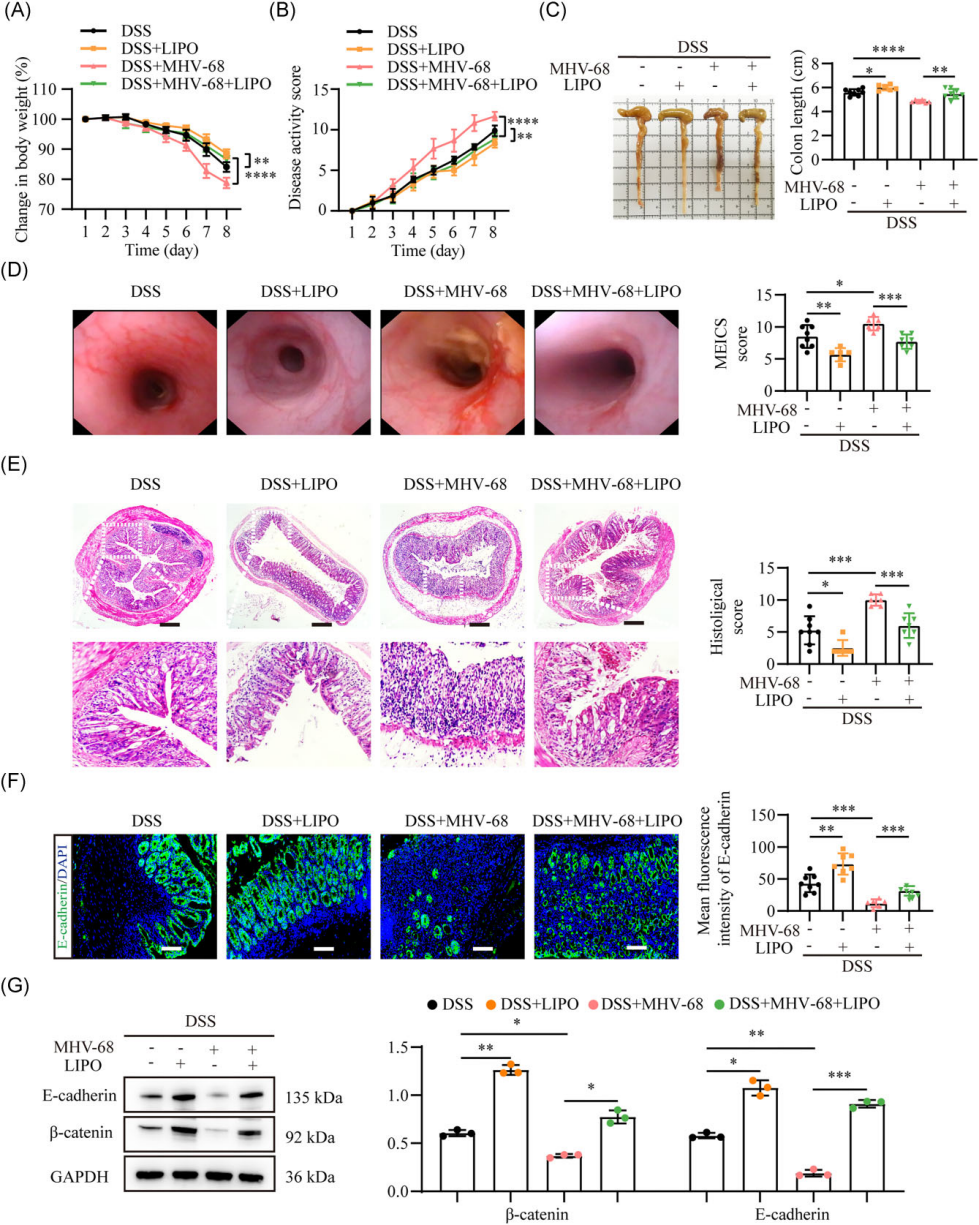
Macrophage depletion alleviates intestinal inflammation exacerbated by MHV-68 infection in DSS-induced colitis. Colitis severity was assessed by weight loss (**A**), DAI score (**B**), representative images of colon length (**C**), mouse endoscopic colitis index of severity (MEICS) score (**D**), and histological scores (**E**) of colonic sections from each group; *n* = 6–8. Immunofluorescence analysis of E-cadherin expression (**F**) in colonic tissues and western blot analysis of colonic E-cadherin and β-catenin protein levels (**G**). DAPI (blue) was used to visualize the nuclei. Scale bars represent 500 μm (E) and 100 μm (F). *P ≤ 0.05, **P ≤ 0.01, ***P ≤ 0.001, ^****^P ≤ 0.0001.

For further confirmation *in vivo*, we intraperitoneally administered LIPO to MHV-68-infected mice 1 day before and 3 days after DSS administration to deplete macrophages. After completing the modeling, we isolated LPMC and used flow cytometry to assess the CD11b⁺F4/80⁺ macrophage count in the colon. Our data confirmed that LIPO treatment effectively reduced macrophage infiltration in the colonic area of mice (supplementary Fig. 6, see online supplementary material). In addition, mice treated with LIPO showed significantly lower levels of weight loss, smaller colons, and a reduced DAI score under MHV-68 infection compared to PBS-treated mice (Fig. 5A–C). Endoscopic and histological evaluations showed that LIPO treatment significantly alleviated the structural injury and colonic inflammation induced by MHV-68 infection in colitis (Fig. 5D and E). Additionally, LIPO treatment improved colonic barrier integrity by preventing the downregulation of β-catenin and E-cadherin expression (Fig. 5F and G). In summary, LIPO administration significantly alleviates the detrimental effects of MHV-68 on classical signs of colitis, highlighting the essential role of macrophages in MHV-68-mediated intestinal inflammation.

### MHV-68-induced macrophage pyroptosis is associated with upregulation of glycolysis

**Figure 6.**
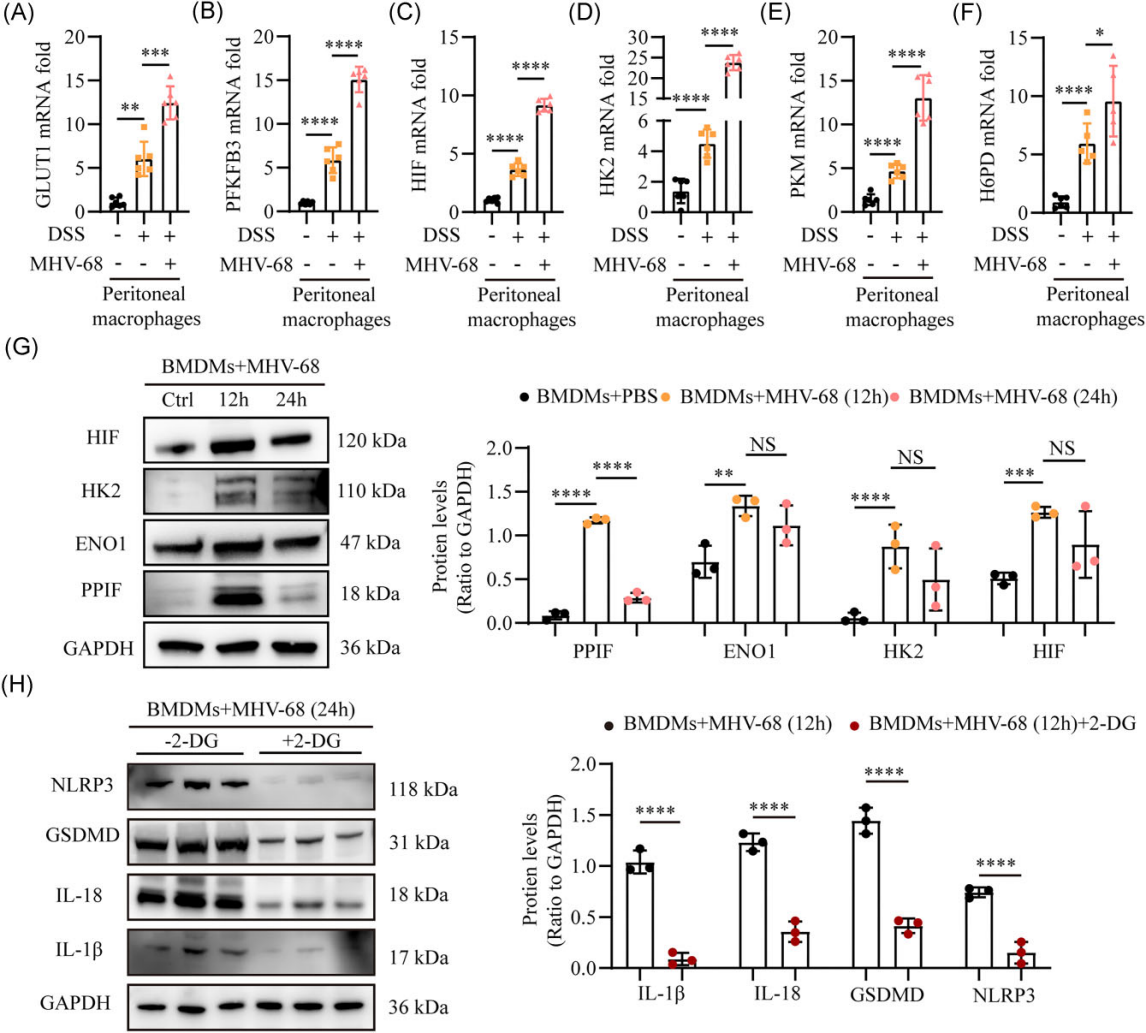
MHV-68-induced macrophage pyroptosis is associated with the upregulation of glycolysis. Relative mRNA levels of glycolysis-related genes, including GLUT1 (**A**), PFKFB3 (**B**), HIF (**C**), HK2 (**D**), PKM (**E**), and H6PD (**F**), were measured in peritoneal macrophages isolated from control mice, mice co-treated with DSS and MHV-68, and mice treated with DSS alone, and normalized to GAPDH. (**G**) Protein levels of glycolysis-related markers, including HIF, HK2, ENO1, and PPIF, were assessed in BMDMs challenged with MHV-68. (**H**) Protein levels of pyroptosis markers, including NLRP3, GSDMD, IL-18, and IL-1β, were measured by western blotting in MHV-68-induced BMDMs treated with or without the glycolysis inhibitor 2-DG. *P ≤ 0.05, **P ≤ 0.01, ***P ≤ 0.001, and ^****^P ≤ 0.0001. NS, Not significant.

Glycolysis, the metabolic pathway that converts glucose into pyruvate, is pivotal for modulating the activity of immune cells and orchestrating inflammatory processes [40]. Available data highlight that EBV infection can induce upregulation of glycolysis in macrophages [41], and enhanced glycolysis in macrophages has been linked to the induction of pyroptosis [42]. Therefore, we hypothesized that EBV promotes pyroptosis by enhancing glycolysis in macrophages. To test our hypothesis, RT-qPCR was used to quantify the expression levels of genes involved in glycolysis, including glucose transporter 1 (GLUT1), 6-phosphofructo-2-kinase/fructose-2,6-bisphosphatase 3 (PFKFB3), hypoxia-inducible factor (HIF), hexokinase 2 (HK2), hexose-6-phosphate dehydrogenase (H6PD), and pyruvate kinase M (PKM), in peritoneal macrophages isolated from mice co-treated with DSS and MHV-68, as well as from mice treated with DSS alone. Significant upregulation of these genes was recorded in mice undergoing dual treatments compared to those treated with DSS alone (Fig. 6A–F). Simultaneously, we analyzed the protein levels of glycolysis markers, including HIF, HK2, enolase 1 (ENO1), and peptidylprolyl isomerase F (PPIF) in BMDMs stimulated with MHV-68. This analysis demonstrated significant increases in these markers at 12 h in the BMDMs with MHV-68 infection, as opposed to the group without MHV-68 infection (Fig. 6G). The evidence indicated that MHV-68 enhanced glycolysis in macrophages, as demonstrated by studies conducted both *in vivo* and *in vitro*. To further ascertain whether MHV-68-induced glycolysis promotes macrophage pyroptosis, we treated MHV-68-induced BMDMs with the glycolysis inhibitor 2-DG. As expected, an apparent decrease in IL-1β, IL-18, NLRP3, and GSDMD expression was observed in macrophages induced by MHV-68 when glycolysis was inhibited (Fig. 6H). These results reveal that MHV-68 infection upregulates glycolysis to induce macrophage pyroptosis, whereas glycolysis inhibition attenuates MHV-68-induced pyroptosis.

## Discussion

**Figure 7.**
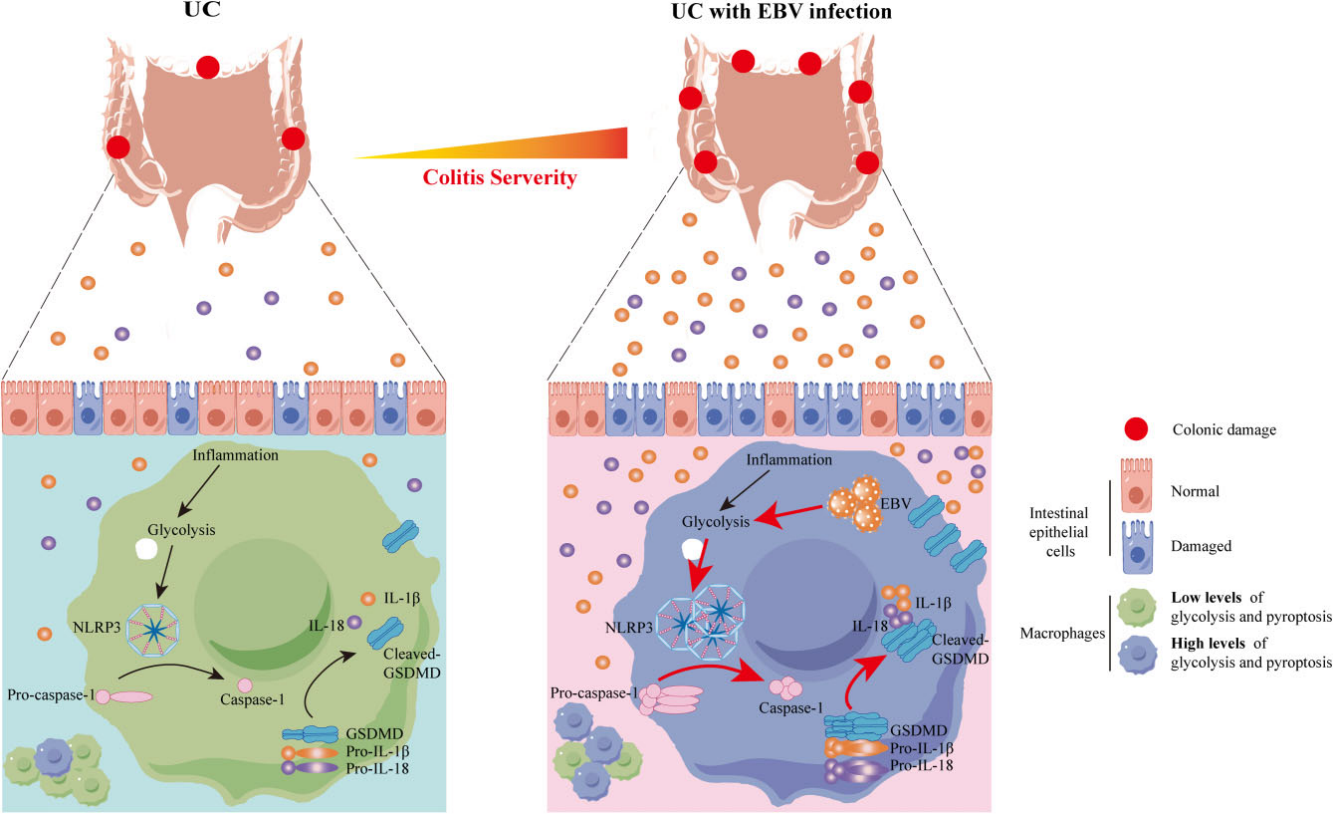
Mechanistic illustration of EBV-induced glycolysis driving macrophage pyroptosis and exacerbating UC inflammation. EBV infection induces the upregulation of glycolysis in intestinal macrophages, which subsequently activates gasdermin D, NLRP3, IL-1β, and IL-18 in macrophages within colonic tissues. The release of these pro-inflammatory cytokines results in intestinal barrier dysfunction and exacerbates UC.

Although the close association between EBV infection and UC has been well-documented in clinical practice, the underlying mechanisms by which EBV exacerbates UC inflammation remain largely unknown. By integrating clinical observations with experimental findings, we provide comprehensive insights into the mechanisms by which EBV drives UC pathogenesis by promoting macrophage pyroptosis (Fig. 7).

The relationship between EBV infection and disease activity in IBD remains a central but incompletely understood topic in research. Recent studies have shown that the introduction of EBV DNA into DSS-induced experimental colitis models significantly exacerbates colonic inflammation, suggesting a role of EBV DNA in enhancing intestinal inflammation [43, 44]. However, the use of EBV DNA alone does not fully capture the complexities of live viral infection in the context of intestinal inflammation. In contrast, our study introduces a novel model using live MHV-68 to infect DSS-treated mice, providing a more accurate representation of EBV infection in IBD patients. Thus, our findings provide strong evidence that MHV-68 infection significantly worsens colonic inflammation in mice with DSS-induced colitis, offering unusual viewpoints on the involvement of EBV in IBD pathogenesis. However, it is important to note that despite some immunological similarities between MHV-68 and EBV, their infection dynamics and mechanisms may differ, necessitating further validation of our findings in human clinical studies.

Macrophages are the most frequently recruited inflammatory cells within the intestinal microenvironment of UC, and their overactivation has been consistently observed during the development of UC [45–47]. The participation of EBV in macrophage-mediated pathogenesis has been demonstrated in several other diseases, including nasopharyngeal carcinoma and multiple sclerosis [48]. However, whether EBV exacerbates UC through the immunomodulation of intestinal macrophages remains largely unknown. Recently, it was reported that EBV is present in LPMCs derived from the inflamed gut mucosa of UC patients, particularly among regions exhibiting severe ulceration and deep inflammation, pointing to the possibility that EBV infection facilitates the initiation of chronic mucosal damage in UC by way of its effects on intestinal macrophages [49, 50]. In this study, we performed adoptive transfer of MHV-68-induced macrophages into mice with DSS-induced colitis, and depleted macrophages in MHV-68-infected mice with colitis. Our findings showed that adoptive transfer of MHV-68-induced macrophages aggravated DSS-induced colitis, while macrophage depletion attenuated the exacerbation of intestinal inflammation induced by MHV-68. These results elucidate the pivotal roles of macrophages in MHV-68-induced intestinal inflammation in colitis. It is worth noting that EBV predominantly infects epithelial cells and B lymphocytes, while our study particularly targeted the modulation of EBV infection on intestinal macrophages [51]. Future investigations should explore how EBV interacts with different cell types, including epithelial cells, dendritic cells, and T lymphocytes, to better understand its effects on intestinal inflammation and immune dysregulation.

Extensive research has been conducted to clarify the role of pyroptosis in modulating the inflammatory responses in UC, but its functions remain controversial. On one hand, absence of NLRP3 inflammasomes and caspase-1 has been shown to increase the inclination of mice towards developing colitis in response to DSS, even leading to mortality [52]. Additionally, macrophage-specific deletion of GSDMD exacerbates colitis by enhancing cyclic GMP-AMP synthase-mediated inflammatory responses [53]. These findings suggested that pyroptosis may protect against UC. Conversely, additional studies have suggested that an overabundance of pyroptosis can worsen colitis. For instance, the experimental colitis induced by DSS or 2,4,6-trinitrobenzenesulfonic acid was significantly alleviated by either knocking out NLRP3 and caspase-1 genes or administering pyroptosis inhibitors [21]. Furthermore, increased numbers of IL-1β^+^ macrophages and monocytes are evident in the colonic tissues of patients with UC [16]. In this study, it was established that EBV infection caused a notable augmentation of pyroptosis markers (IL-1β, IL-18, GSDMD, and NLRP3) in the colonic macrophages of patients with UC, which was positively correlated with disease severity. Specifically, the strong correlations between CD68⁺IL-18⁺ and CD68⁺IL-1β⁺ macrophages and disease activity markers (*R*² values ranging from 0.6453 to 0.8261) highlight the contribution of IL-18 and IL-1β released during pyroptosis to systemic inflammation and disease severity. Interestingly, while the correlation coefficients for CD68⁺GSDMD⁺ macrophages were slightly lower, the significance observed for several markers (e.g. platelet count and Mayo Clinic Activity Score) underscores the critical role of GSDMD as an upstream effector of the pyroptosis pathway. Similarly, in DSS-induced colitis, MHV-68 infection resulted in significant expression of GSDMD, NLRP3, IL-1β, and IL-18 in peritoneal macrophages. *In vitro* experiments further demonstrated that EBV and MHV-68 can directly induce pyroptosis in human THP-1 macrophages and murine BMDMs, respectively. Our study revealed that intestinal EBV infection exacerbates colonic inflammation in UC through activation of macrophage pyroptosis. These findings are consistent with previous research showing that pathogenic infection exacerbated DSS-induced colitis by elevating IL-1β levels in intestinal macrophages [54]. Taken together, our results reinforce the notion that heightened activation of pyroptotic signaling pathways in macrophages exacerbates colonic inflammation in UC. However, the precise mechanisms by which EBV-induced macrophage pyroptosis mediates intestinal inflammation in colitis remain unclear and warrant further investigation.

Mounting evidence underscores that glycolysis is a core regulator of pyroptosis in inflammatory diseases and cancers [55]. It is shown that glycolysis inhibitors not only suppressed the expression of GSDMD in lipopolysaccharide-induced macrophage pyroptosis *in vitro* but also attenuated pyroptosis of murine macrophages *in vivo* [56]. In nasopharyngeal carcinoma triggered by EBV infection, EBV-encoded latent membrane protein 1 directly interacted with the glycolytic protein Glut1 to induce NLRP3 and IL-1β expression in myeloid-derived suppressor cells [12, 41]. Consistent with these findings, our study proposes glycolysis as a viable therapeutic target to counteract EBV-induced macrophage pyroptosis in colitis. We demonstrated that MHV-68-induced glycolysis correlated with increased pyroptosis in murine macrophages, and inhibition of glycolysis attenuated pyroptosis *in vitro*. This suggests that targeting glycolytic pathways may offer therapeutic benefits for MHV-68-driven intestinal inflammation. However, whether EBV induces macrophage pyroptosis by activating glycolysis requires further investigation in UC patients. Specifically, verifying the interaction between pyroptosis and glycolysis through co-immunoprecipitation and validating their upstream and downstream relationships in animal models is necessary.

Our study has certain limitations. Firstly, we did not directly use EBV-treated CD14⁺ cells from human PBMCs to confirm the effects of EBV on macrophage pyroptosis. Although our experiments using THP-1 cells provided valuable insights, future studies utilizing primary human macrophages, such as CD14⁺ cells, are warranted to further validate the role of EBV in inducing pyroptosis in human macrophages. Secondly, our study did not incorporate the use of pyroptosis inhibitors, such as the NLRP3 inflammasome inhibitor MCC950, to assess whether the suppression of pyroptosis could mitigate MHV-68-induced colitis. While the macrophage depletion model employed in our research provided specific evidence for the role of macrophage pyroptosis in intestinal inflammation, future studies will prioritize the use of pyroptosis inhibitors to further elucidate the underlying mechanisms of EBV-aggravated colitis and explore potential therapeutic interventions. Thirdly, our study did not quantitatively assess the MHV-68 viral load in colonic tissues using RT-qPCR. This limitation does not diminish the significance of our findings but highlights the need for future studies to develop robust quantitative methods to further validate the extent of MHV-68 infection in intestinal tissues. Lastly, while this study focused on macrophages as a key mediator of EBV-induced intestinal inflammation, other immune cells, such as neutrophils and lymphocytes, may also play critical roles in the pathogenesis of EBV-associated colitis. Future studies should investigate the roles of neutrophils and lymphocytes in EBV-associated colitis to provide a more comprehensive understanding of the immune landscape of this disease.

In conclusion, our findings elucidate a mechanistic link between EBV infection and the exacerbation of UC through the activation of macrophage pyroptosis. This novel insight offers a potential avenue for mitigating intestinal inflammatory responses associated with EBV infection.

## Supplementary Material

pbaf002_Supplemental_File

## Data Availability

All relevant data are contained within the article and the supplementary material. Additional data supporting the findings of this article are available from the corresponding author upon reasonable request.

## References

[1] Zhang Y, Chu X, Wang L et al. Global patterns in the epidemiology, cancer risk, and surgical implications of inflammatory bowel disease. Gastroenterol Rep (Oxf). 2024;12:goae053. 10.1093/gastro/goae053. 38984068 PMC11233070

[2] Zhu L, Xie Z, Yang G et al. Stanniocalcin-1 promotes PARP1-dependent cell death via JNK activation in colitis. Adv Sci (Weinh). 2024;11:e2304123. 10.1002/advs.202304123. 38088577 PMC10837357

[3] Raine T, Bonovas S, Burisch J et al. ECCO Guidelines on therapeutics in ulcerative colitis: medical treatment. J Crohns Colitis. 2022;16:2–17. 10.1093/ecco-jcc/jjab178. 34635919

[4] Li L, Cheng R, Wu Y et al. Diagnosis and management of inflammatory bowel disease. J Evid Based Med. 2024;17:409–33. 10.1111/jebm.12626. 38934234

[5] Levhar N, Ungar B, Kopylov U et al. Propagation of EBV-driven lymphomatous transformation of peripheral blood B cells by immunomodulators and biologics used in the treatment of inflammatory bowel disease. Inflamm Bowel Dis. 2020;26:1330–39. 10.1093/ibd/izaa065. 32322878

[6] Zhang Y, Tian F, Li H Pulmonary infection due to reactivation of latent Epstein-Barr virus in a patient with Crohn's disease treated with Infliximab. Inflamm Bowel Dis. 2022;28:e80–1. 10.1093/ibd/izab304. 35657372

[7] Dang Y, Ma C, Chen K et al. The effects of a high-fat diet on inflammatory bowel disease. Biomolecules. 2023;13:905. 10.3390/biom13060905. 37371485 PMC10296751

[8] Ciccocioppo R, Racca F, Paolucci S et al. Human cytomegalovirus and Epstein-Barr virus infection in inflammatory bowel disease: need for mucosal viral load measurement. World J Gastroenterol. 2015;21:1915–26. 10.3748/wjg.v21.i6.1915. 25684960 PMC4323471

[9] Hosomi S, Watanabe K, Nishida Y et al. Combined infection of Human Herpes viruses: A risk factor for subsequent colectomy in ulcerative colitis. Inflamm Bowel Dis. 2018;24:1307–15. 10.1093/ibd/izy005. 29668948

[10] Xu S, Chen H, Zu X et al. Epstein-Barr virus infection in ulcerative colitis: a clinicopathologic study from a Chinese area. Therap Adv Gastroenterol. 2020;13:1756284820930124. 10.1177/1756284820930124. PMC7444145 32913442

[11] Li X, Chen N, You P et al. The status of Epstein-Barr Virus infection in intestinal mucosa of Chinese patients with inflammatory bowel disease. Digestion. 2019;99:126–32. 10.1159/000489996. 30235444

[12] Ma C, Jiang MS, Li JX et al. Plasma Epstein-Barr Virus DNA load for diagnostic and prognostic assessment in intestinal Epstein-Barr Virus infection. Front Cell Infect Microbiol. 2024;14:1526633. 10.3389/fcimb.2024.1526633. 39839261 PMC11747383

[13] Wang Z, Zhang W, Luo C et al. Primary intestinal Epstein-Barr Virus-associated natural killer/T-cell lymphoproliferative disorder: A disease mimicking inflammatory bowel disease. J Crohns Colitis. 2018;12:896–904. 10.1093/ecco-jcc/jjy043. 29635312

[14] Yuan YY, Xie KX, Wang SL et al. Inflammatory caspase-related pyroptosis: mechanism, regulation and therapeutic potential for inflammatory bowel disease. Gastroenterol Rep (Oxf). 2018;6:167–76. 10.1093/gastro/goy011. 30151200 PMC6101557

[15] Man SM Inflammasomes in the gastrointestinal tract: infection, cancer and gut microbiota homeostasis. Nat Rev Gastroenterol Hepatol. 2018;15:721–37. 10.1038/s41575-018-0054-1. 30185915 PMC7097092

[16] Zhen Y, Zhang H NLRP3 Inflammasome and inflammatory bowel disease. Front Immunol. 2019;10:276. 10.3389/fimmu.2019.00276. 30873162 PMC6403142

[17] Gong W, Liu P, Zhao F et al. STING-mediated syk signaling attenuates tumorigenesis of colitis–associated colorectal cancer through enhancing intestinal epithelium pyroptosis. Inflamm Bowel Dis. 2022;28:572–85. 10.1093/ibd/izab217. 34473281

[18] Liu H, Irwanto A, Tian H et al. Identification of IL18RAP/IL18R1 and IL12B as leprosy risk genes demonstrates shared pathogenesis between inflammation and infectious diseases. Am J Hum Genet. 2012;91:935–41. 10.1016/j.ajhg.2012.09.010. 23103228 PMC3487119

[19] Hanaei S, Sadr M, Rezaei A et al. Association of NLRP3 single nucleotide polymorphisms with ulcerative colitis: A case-control study. Clin Res Hepatol Gastroenterol. 2018;42:269–75. 10.1016/j.clinre.2017.09.003. 29102545

[20] Wu J, Zhang X, Wu D et al. Evaluation of causal associations between interleukin-18 levels and immune-mediated inflammatory diseases: a Mendelian randomization study. BMC Med Genomics. 2023;16:306. 10.1186/s12920-023-01744-z. 38031150 PMC10685486

[21] Perera AP, Fernando R, Shinde T et al. MCC950, a specific small molecule inhibitor of NLRP3 inflammasome attenuates colonic inflammation in spontaneous colitis mice. Sci Rep. 2018;8:8618. 10.1038/s41598-018-26775-w. 29872077 PMC5988655

[22] Zeng B, Huang Y, Chen S et al. Dextran sodium sulfate potentiates NLRP3 inflammasome activation by modulating the KCa3.1 potassium channel in a mouse model of colitis. Cell Mol Immunol. 2022;19:925–43. 10.1038/s41423-022-00891-0. 35799057 PMC9338299

[23] Yang W, Liu H, Xu L et al. GPR120 Inhibits colitis through regulation of CD4(+) T cell interleukin 10 production. Gastroenterology. 2022;162:150–65. 10.1053/j.gastro.2021.09.018. 34536451 PMC8678294

[24] Weber-Stiehl S, Taubenheim J, Järke L et al. Hexokinase 2 expression in apical enterocytes correlates with inflammation severity in patients with inflammatory bowel disease. BMC Med. 2024;22:490. 10.1186/s12916-024-03710-7. 39444028 PMC11515617

[25] Lv Q, Xing Y, Liu Y et al. Didymin switches M1-like toward M2-like macrophage to ameliorate ulcerative colitis via fatty acid oxidation. Pharmacol Res. 2021;169:105613. 10.1016/j.phrs.2021.105613. 33915297

[26] Song W, Gao Y, Wu J et al. LMP1 enhances aerobic glycolysis in natural killer/T cell lymphoma. Cell Death Dis. 2024;15:604. 10.1038/s41419-024-06999-7. 39164228 PMC11335758

[27] Lyu X, Wang J, Guo X et al. EBV-miR-BART1-5P activates AMPK/mTOR/HIF1 pathway via a PTEN independent manner to promote glycolysis and angiogenesis in nasopharyngeal carcinoma. PLoS Pathog. 2018;14:e1007484. 10.1371/journal.ppat.1007484. 30557400 PMC6312352

[28] Lo AK, Dawson CW, Young LS et al. Activation of the FGFR1 signalling pathway by the Epstein-Barr virus-encoded LMP1 promotes aerobic glycolysis and transformation of human nasopharyngeal epithelial cells. J Pathol. 2015;237:238–48. 10.1002/path.4575. 26096068

[29] Weber S, Sitte S, Voegele AL et al. NLRP3 Inhibition leads to impaired mucosal fibroblast function in patients with inflammatory bowel diseases. J Crohns Colitis. 2024;18:446–61. 10.1093/ecco-jcc/jjad164. 37748021

[30] Chen K, Shang S, Yu S et al. Identification and exploration of pharmacological pyroptosis-related biomarkers of ulcerative colitis. Front Immunol. 2022;13:998470. 10.3389/fimmu.2022.998470. 36311726 PMC9606687

[31] Van de Veerdonk FL, Wever PC, Hermans MH et al. IL-18 serum concentration is markedly elevated in acute EBV infection and can serve as a marker for disease severity. J Infect Dis. 2012;206:197–201. 10.1093/infdis/jis335. 22689912

[32] Kianfar R, Ravanshad M, Ghiass MA et al. Evaluation of IL-1β and IL-6 expression following EBNA-1 and BRLF-1 peptide treatment in Epstein-Barr Virus-positive Multiple sclerosis patients. Intervirology. 2022;65:144–50. 10.1159/000522577. 35158367 PMC9501781

[33] Torii Y, Kawada JI, Murata T et al. Epstein-Barr virus infection-induced inflammasome activation in human monocytes. PLoS One. 2017;12:e0175053. 10.1371/journal.pone.0175053. 28369146 PMC5378412

[34] Wirtz S, Popp V, Kindermann M et al. Chemically induced mouse models of acute and chronic intestinal inflammation. Nat Protoc. 2017;12:1295–309. 10.1038/nprot.2017.044. 28569761

[35] Rachmilewitz D, Karmeli F, Takabayashi K et al. Immunostimulatory DNA ameliorates experimental and spontaneous murine colitis. Gastroenterology. 2002;122:1428–41. 10.1053/gast.2002.32994. 11984528

[36] Becker C, Fantini MC, Neurath MF High resolution colonoscopy in live mice. Nat Protoc. 2006;1:2900–4. 10.1038/nprot.2006.446. 17406549

[37] Ye C, Wang A, Li W et al. Prealbumin as a prognostic indicator for hospital readmission of ulcerative colitis patients. Precis Clin Med. 2024;7:pbad026. 10.1093/pcmedi/pbad026. 38196560 PMC10773210

[38] Zeng Z, Jiang M, Li X et al. Precision medicine in inflammatory bowel disease. Precis Clin Med. 2023;6:pbad033. 10.1093/pcmedi/pbad033. 38638127 PMC11025389

[39] Bullard WL, Kara M, Gay LA et al. Identification of murine gammaherpesvirus 68 miRNA-mRNA hybrids reveals miRNA target conservation among gammaherpesviruses including host translation and protein modification machinery. PLoS Pathog. 2019;15:e1007843. 10.1371/journal.ppat.1007843. 31393953 PMC6687095

[40] Zeng Z, Cheng S, Li X et al. Glycolytic activation of CD14+ intestinal macrophages contributes to the inflammatory responses via exosomal membrane tumor necrosis factor in Crohn's Disease. Inflamm Bowel Dis. 2024;30:90–102. 10.1093/ibd/izad117. 37406645

[41] Cai TT, Ye SB, Liu YN et al. LMP1-mediated glycolysis induces myeloid-derived suppressor cell expansion in nasopharyngeal carcinoma. PLoS Pathog. 2017;13:e1006503. 10.1371/journal.ppat.1006503. 28732079 PMC5540616

[42] Uematsu T, Tsuchiya K, Kobayashi N et al. Mint3 depletion-mediated glycolytic and oxidative alterations promote pyroptosis and prevent the spread of Listeria monocytogenes infection in macrophages. Cell Death Dis. 2021;12:404. 10.1038/s41419-021-03691-y. 33854054 PMC8046764

[43] Karout I, Salhab Z, Sherri N et al. The effects of endosomal toll-like receptor inhibitors in an EBV DNA-exacerbated inflammatory bowel disease mouse model. Viruses. 2024;16:624. 10.3390/v16040624. 38675965 PMC11054613

[44] Andari S, Hussein H, Fadlallah S et al. Epstein-Barr virus DNA exacerbates colitis symptoms in a mouse model of inflammatory bowel disease. Viruses. 2021;13:1272. 10.3390/v13071272. 34210024 PMC8310145

[45] Chen L, Li J, Ye Z et al. Anti-high mobility group box 1 neutralizing-antibody ameliorates dextran sodium sulfate colitis in mice. Front Immunol. 2020;11:585094. 10.3389/fimmu.2020.585094. 33193406 PMC7661783

[46] Kang G, Wang X, Gao M et al. Propionate-producing engineered probiotics ameliorated murine ulcerative colitis by restoring anti-inflammatory macrophage via the GPR43/HDAC1/IL-10 axis. Bioeng Transl Med. 2024;9:e10682. 10.1002/btm2.10682. 39553425 PMC11561831

[47] Chen L, Wang Y, Zhou H et al. The new insights of hyperbaric oxygen therapy: focus on inflammatory bowel disease. Precis Clin Med. 2024;7:pbae001. 10.1093/pcmedi/pbae001. 38344218 PMC10858389

[48] Shimakage M Significant role of macrophages in human cancers associated with Epstein-Barr virus (Review). Oncol Rep. 2014;32:1763–71. 10.3892/or.2014.3475. 25224510

[49] Pezhouh MK, Miller JA, Sharma R et al. Refractory inflammatory bowel disease: is there a role for Epstein-Barr virus? A case-controlled study using highly sensitive Epstein-Barr virus-encoded small RNA1 in situ hybridization. Hum Pathol. 2018;82:187–92. 10.1016/j.humpath.2018.08.001. 30120969

[50] Liu R, Wang M, Zhang L et al. The clinicopathologic features of chronic active Epstein-Barr virus infective enteritis. Mod Pathol. 2019;32:387–95. 10.1038/s41379-018-0144-1. 30297882

[51] Young LS, Yap LF, Murray PG Epstein-Barr virus: more than 50 years old and still providing surprises. Nat Rev Cancer. 2016;16:789–02. 10.1038/nrc.2016.92. 27687982

[52] Zaki MH, Boyd KL, Vogel P et al. The NLRP3 inflammasome protects against loss of epithelial integrity and mortality during experimental colitis. Immunity. 2010;32:379–91. 10.1016/j.immuni.2010.03.003. 20303296 PMC2982187

[53] Ma C, Yang D, Wang B et al. Gasdermin D in macrophages restrains colitis by controlling cGAS-mediated inflammation. Sci Adv. 2020;6:eaaz6717. 10.1126/sciadv.aaz6717. 32671214 PMC7314554

[54] Dong D, Su T, Chen W et al. Clostridioides difficile aggravates dextran sulfate solution (DSS)-induced colitis by shaping the gut microbiota and promoting neutrophil recruitment. Gut Microbes. 2023;15:2192478. 10.1080/19490976.2023.2192478. 36951545 PMC10038061

[55] Olona A, Leishman S, Anand PK The NLRP3 inflammasome: regulation by metabolic signals. Trends Immunol. 2022;43:978–89. 10.1016/j.it.2022.10.003. 36371361

[56] He Y, Wang Y, Jia X et al. Glycolytic reprogramming controls periodontitis-associated macrophage pyroptosis via AMPK/SIRT1/NF-κB signaling pathway. Int Immunopharmacol. 2023;119:110192. 10.1016/j.intimp.2023.110192. 37068341

