# Supplementary material for: Epstein–Barr virus infection exacerbates ulcerative colitis by driving macrophage pyroptosis via the upregulation of glycolysis
Source: Precis Clin Med. 2025 Jan 21;8(1):pbaf002. doi: 10.1093/pcmedi/pbaf002 (PMC11878796; doi:10.1093/pcmedi/pbaf002)
Supplement: pbaf002_Supplemental_File [file pbaf002_supplemental_file.docx]

**Supplementary Materials**

**Supplementary Table 1.** Information of antibodies used in this study.

| **Antibody** | **Source** | **Cat#** |
| --- | --- | --- |
| Mouse anti-CD68 antibody | Biolegend | 916104 |
| Rabbit anti-NLRP3 antibody | Cell Signaling Technology | 15101 |
| Rabbit anti-GSDMD antibody | Proteintech | 20770-1-AP |
| Rabbit anti-IL-18 antibody | Proteintech | 10663-1-AP |
| Rabbit anti-IL-1β antibody | Affinity | AF4006 |
| Rabbit anti-E-cadherin antibody | Cell Signaling Technology | 3195 |
| Rabbit anti-β-catenin antibody | Cell Signaling Technology | 9562 |

**Supplementary Table 2.** Primer sequences used for RT-qPCR in this study.

| **Genes** | **Forward primer** | **Reverse primer** |
| --- | --- | --- |
| Human *TNF-α* | CACTTTGGAGTGATCGGC | GCTTGAGGGTTTGCTACAACA |
| Human *IL-6* | CCTTCTCCACAAGCGCCTTC | GGAAGGCAGCAGGCAACA |
| Human *IL-10* | GAAGATGTCAAACTCACTCATGGC | AACAAGAGCAAGGCCGTGG |
| Human *IL-1β* | AGCCATGGCAGAAGTACCTG | CCTGGAAGGAGCACTTCATCT |
| Human *IL-18* | CAGACCTTCCAGATCGCTTC | CCCCCAATTCATCCTCTTTT |
| Human *Caspase1* | GTTTCTTGGAGACATCCC | TAATGTCCTGGGAAGAGG |
| Human *GAPDH* | TGACTTCAACAGCGACACCCA | CACCCTGTTGCTGTAGCCAAA |
| Mouse *TNF-α* | GGTGCCTATGTCTCAGCCTCTT | GCCATAGAACTGATGAGAGGGAG |
| Mouse *IL-6* | AGCCAGAGTCCTTCAGAGAGATAC | AATTGGATGGTCTTGGTCCTTAGC |
| Mouse *IL-10* | GCTCTTACTGACTGGCATGAG | CGCAGCTCTAGGAGCATGTG |
| Mouse *IL-1β* | GCAACTGTTCCTGAACTCAACT | ATCTTTTGGGGTCCGTCAACT |
| Mouse *IL-18* | CCTACTTCAGCATCCTCTACTGG | AGGGTTTCTTGAGAAGGGGAC |
| Mouse *Caspase1* | ACAAGGCACGGGACCTATG | TCCCAGTCAGTCCTGGAAATG |
| Mouse *Glut1* | GCAGTTCGGCTATAACACTGG | GCGGTGGTTCCATGTTTGATTG |
| Mouse *H6PD* | AAGATGCTCCTAGCGGCAATG | TCCAGGTATAGCTGAAACAGTCC |
| Mouse *PFKFB3* | CAACTCCCCAACCGTGATTGT | GAGGTAGCGAGTCAGCTTCTT |
| Mouse *HK2* | ATGATCGCCTGCTTATTCACG | CGCCTAGAAATCTCCAGAAGGG |
| Mouse *HIF-1α* | GATGACGGCGACATGGTTTAC | CTCACTGGGCCATTTCTGTGT |
| Mouse *PKM* | CGCCTGGACATTGACTCTG | GAAATTCAGCCGAGCCACATT |
| Mouse *GAPDH* | TGGCCTTCCGTGTTCCTAC | GAGTTGCTGTTGAAGTCGCA |
| *EBER-1* | CATCCCAGAAGATGCACGCT | TAGCGGACAAGCCGAATACC |
| *EBER-2* | CATCCCAGAAGATGCACGCT | AAGCCTCTCTTCTCCTCCCC |
| *EBER-3* | CATCCCAGAAGATGCACGCT | AGCCGAATACCCTTCTCCCA |
| *EBER-4* | TCCCAGAAGATGCACGCTTA | TAGCGGACAAGCCGAATACC |
| *EBER-5* | CATCCCAGAAGATGCACGCT | GCAAATGCTCTAGGCGGGAA |
| *M1* | ATCTCACCTTTGCTGGATTCTTATTTGC | GTTCTGATGGCTTGAAACGATGGC |

**Supplementary Table 3.** Clinical characteristics of UC patients.

| Characteristics | UC patients  (N=6) | UC patients with EBV infection  (N=5) |
| --- | --- | --- |
| Age (year), median ± SD | 47±9 | 45±18 |
| Male, n (%) | 3 (50%) | 3 (60%) |
| Disease duration (months), median ± SD | 33±37 | 46±53 |
| Fever, n (%) | 1 (16.7%) | 3 (60%) |
| Abdominal pain, n (%) | 4 (66.7%) | 5 (100%) |
| Haematochezia, n (%) | 5 (83.3%) | 4 (80%) |
| Diarrhoea, n (%) | 5 (83.3%) | 5 (100%) |
| Weight loss, n (%) | 4 (66.7%) | 4 (80%) |
| The count of EBER-positive cells per high-power field in colon | 0 | 5-10 |
| Plasma EBV DNA load, median±SD (Copies/mL) | 0±4.94×10^0^ | 8.52×10^1^±8.6×10^1^ |

SD, standard deviation, N, number.


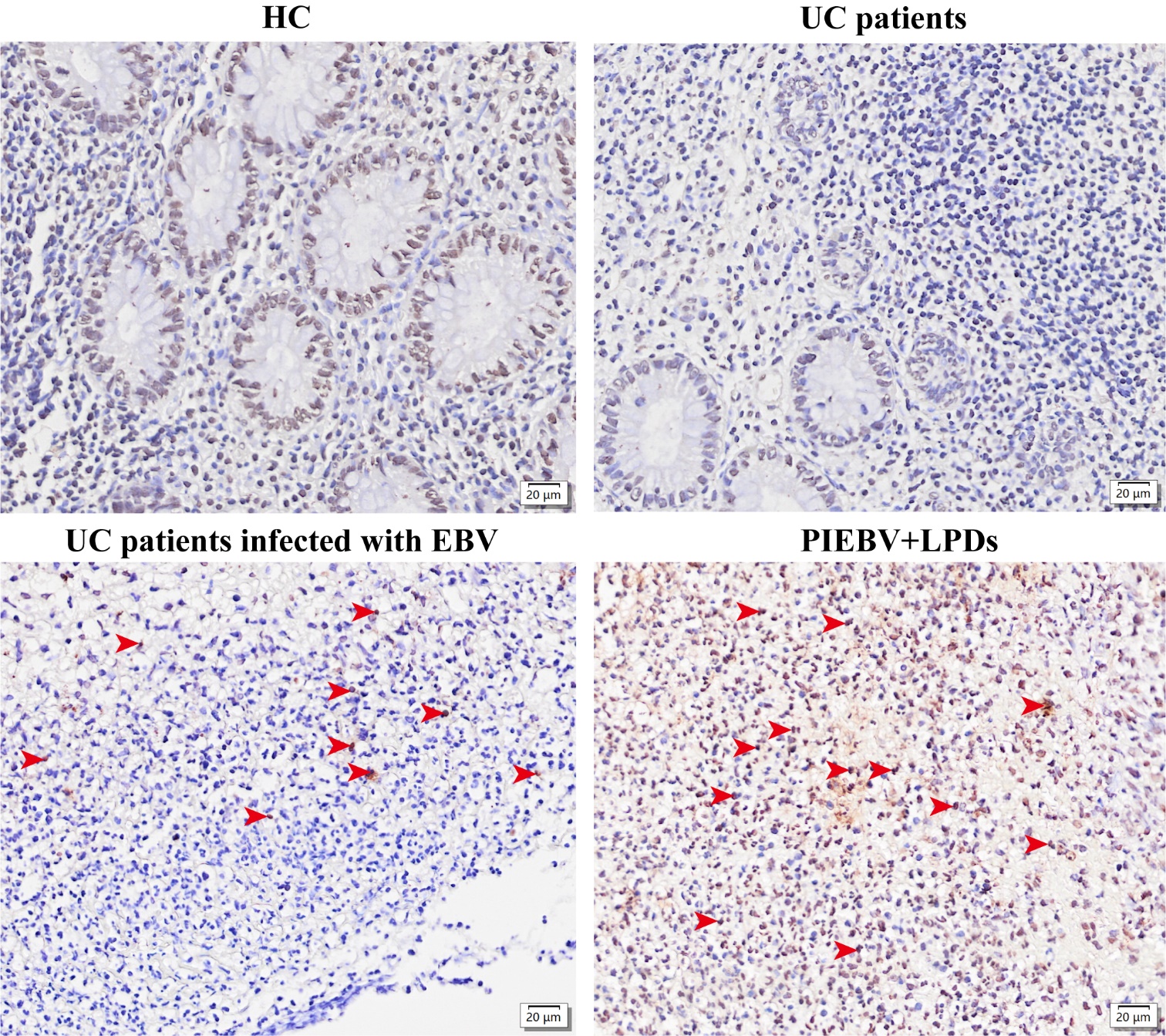


**Supplementary Figure 1. Detection of EBV infection in colon by EBER-ISH.** Representative images of EBER-ISH staining in colonic biopsies from (A) healthy controls (HC), (B) UC patients, (C) UC patients infected with EBV, and (D) patients with primary intestinal EBV-positive lymphoproliferative disorders (PIEBV+LPDs).


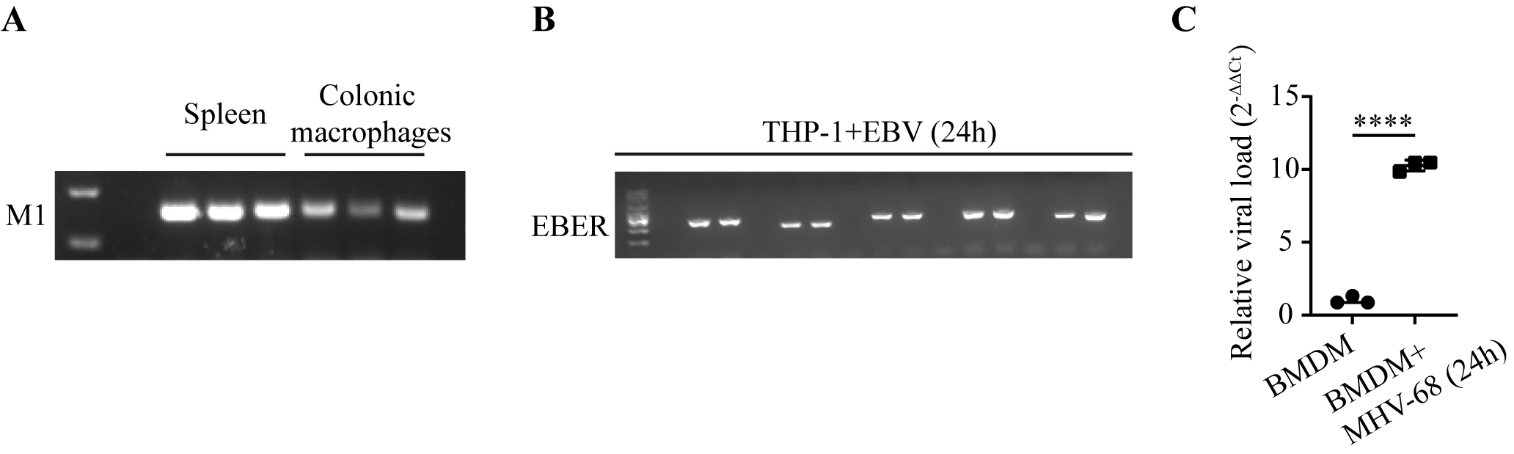


**Supplementary Figure 2. Confirmation of EBV and MHV-68 infection in macrophages both in vivo and in vitro.** (A) DNA-PCR detection of M1 gene in the spleen and colonic macrophages of MHV-68-infected mice. (B) DNA-PCR detection of EBER gene in THP-1 cells following 24 hours of EBV infection. (C) Relative viral load in BMDMs with or without MHV-68 infection for 24 hours.


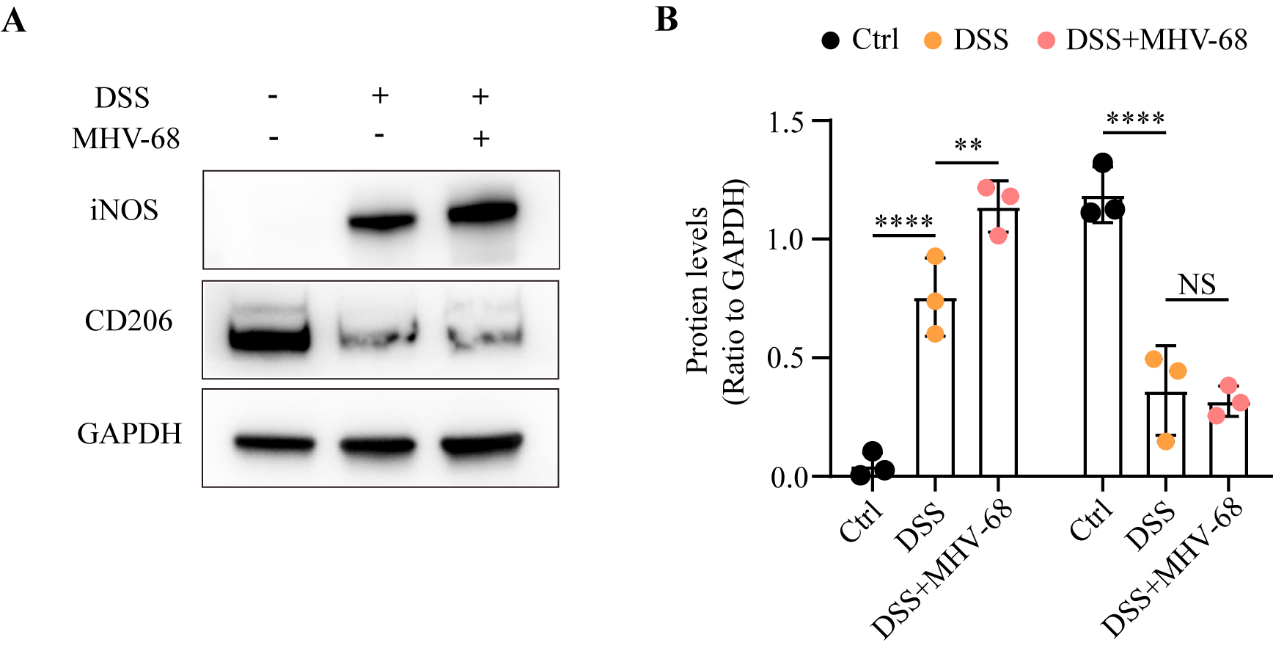


**Supplementary Figure 3. MHV-68 infection promotes M1 macrophage polarization in DSS-induced colitis.** (A) Western blot analysis of iNOS (M1 marker) and CD206 (M2 marker) protein levels in colonic tissues from control (Ctrl), DSS-treated, and DSS+MHV-68-infected mice. (B) Quantification of iNOS and CD206 protein levels relative to GAPDH in colonic tissues. **P < 0.01, ****P < 0.0001, NS, not significant.


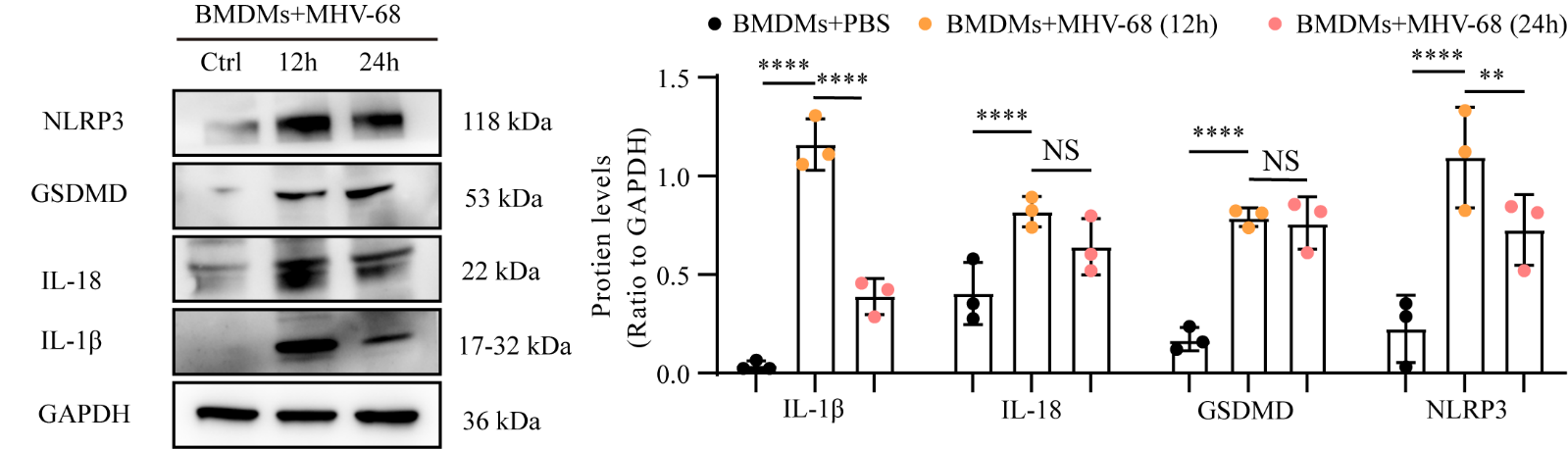


**Supplementary Figure 4.** Western blot analysis of pyroptosis-related protein levels in BMDMs stimulated with MHV-68 for 12 and 24 hours.


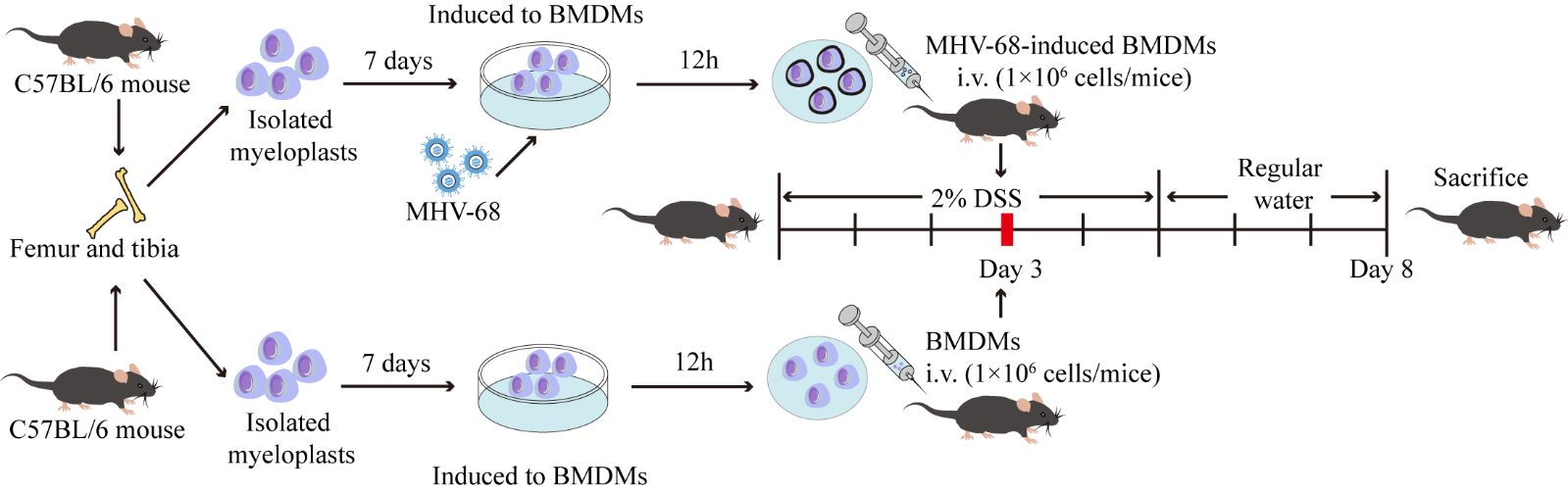


**Supplementary Figure 5.** Schematic diagram depicting the adoptive transfer of MHV-68-induced BMDMs into DSS-induced colitis mice.


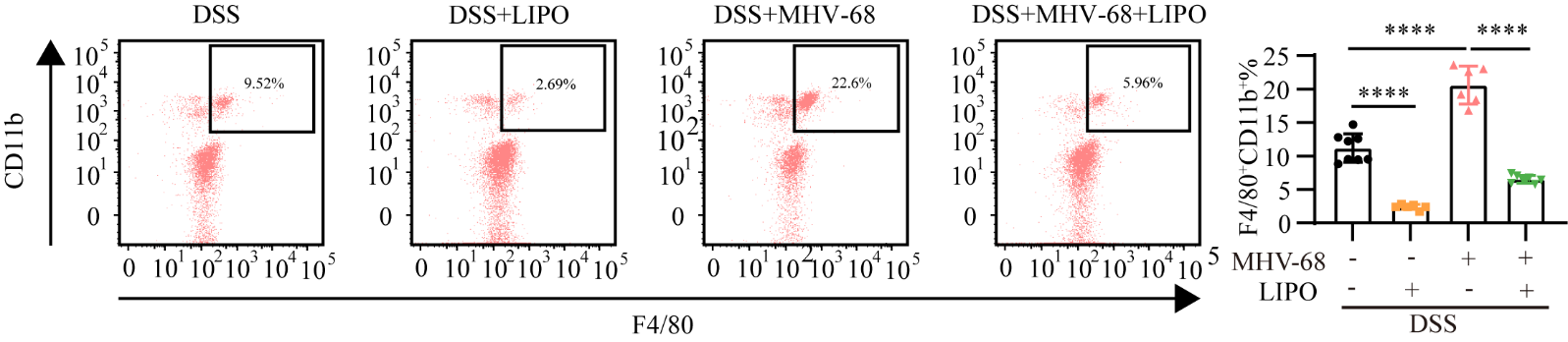


**Supplementary Figure 6.** Flow cytometry analysis of CD11b^+^F4/80^+^ macrophages in the colons of mice treated with or without LIPO.
